# The VINE complex is an endosomal VPS9-domain GEF and SNX-BAR coat

**DOI:** 10.7554/eLife.77035

**Published:** 2022-08-08

**Authors:** Shawn P Shortill, Mia S Frier, Ponthakorn Wongsangaroonsri, Michael Davey, Elizabeth Conibear

**Affiliations:** 1 Department of Medical Genetics, University of British Columbia Vancouver Canada; 2 Centre for Molecular Medicine and Therapeutics, British Columbia Children’s Hospital Research Institute, University of British Columbia Vancouver Canada; Stanford University School of Medicine United States; Stanford University School of Medicine United States

**Keywords:** Vrl1, VINE, Ykr078w, Vps5, retromer, Vps501, *S. cerevisiae*

## Abstract

Membrane trafficking pathways perform important roles in establishing and maintaining the endosomal network. Retrograde protein sorting from the endosome is promoted by conserved SNX-BAR-containing coat complexes including retromer which enrich cargo at tubular microdomains and generate transport carriers. In metazoans, retromer cooperates with VARP, a conserved VPS9-domain GEF, to direct an endosomal recycling pathway. The function of the yeast VARP homolog Vrl1 has been overlooked due to an inactivating mutation found in commonly studied strains. Here, we demonstrate that Vrl1 has features of a SNX-BAR coat protein and forms an obligate complex with Vin1, the paralog of the retromer SNX-BAR protein Vps5. Unique features in the Vin1 N-terminus allow Vrl1 to distinguish it from Vps5, thereby forming a complex that we have named VINE. The VINE complex occupies endosomal tubules and redistributes a conserved mannose 6-phosphate receptor-like protein from endosomes. We also find that membrane recruitment by Vin1 is essential for Vrl1 GEF activity, suggesting that VINE is a multifunctional coat complex that regulates trafficking and signaling events at the endosome.

## eLife digest

All healthy cells have a highly organized interior: different compartments with specialized roles are in different places, and in order to do their jobs properly, proteins need to be in the right place. Endosomes are membrane-bound compartments that act as transport hubs where proteins are sorted into small vesicles and delivered to other parts of the cell. Two groups of proteins regulate this transport: the first group, known as VPS9 GEFs, switches on the enzymes that recruit the second group of proteins, called the sorting nexins. This second group is responsible for forming the transport vesicles via which proteins are distributed all over the cell. Defects in protein sorting can lead to various diseases, including neurodegenerative conditions such as Parkinson’s disease and juvenile amyotrophic lateral sclerosis.

Scientists often use budding yeast cells to study protein sorting, because these cells are similar to human cells, but easier to grow in large numbers and examine in the laboratory. Previous work showed that a yeast protein called Vrl1 is equivalent to a VPS9 GEF from humans called VARP. However, Vrl1 only exists in wild forms of budding yeast, and not in laboratory strains of the organism. Therefore, researchers had not studied Vrl1 in detail, and its roles remained unclear.

To learn more about Vrl1, Shortill et al. started by re-introducing the protein into laboratory strains of budding yeast and observing what happened to protein sorting in these cells. Like VARP, Vrl1 was found in the endosomes of budding yeast. However, biochemical experiments revealed that, while human VARP binds to a protein called retromer, Vrl1 does not bind to the equivalent protein in yeast. Instead, Vrl1 itself has features of both the VPS9 GEFs and the sorting nexins. Shortill et al. also found that Vrl1 interacted with a different protein in the sorting nexin family called Vin1. In the absence of Vrl1, Vin1 was found floating around the cell, but once Vrl1 was re-introduced into the budding yeast, Vin1 relocated to the endosomes. Vrl1 uses its VPS9 GEF part to move itself to the endosome membrane, and Vin1 controls this movement, highlighting the interdependence between the two proteins. Once they are at the endosome together, Vrl1 and Vin1 help redistribute proteins to other parts of the cell.

This study suggests that, like VARP, Vrl1 cooperates with sorting nexins to transport proteins. Since many previous experiments about protein sorting were carried out in yeast cells lacking Vrl1, it is possible that this process was overlooked despite its potential importance. These new findings could also help other researchers investigating how endosomes and protein sorting work, or do not work, in the context of neurodegenerative diseases.

## Introduction

Transport of proteins and lipids at the endosome requires the concerted action of peripheral cargo-sorting complexes, Rab-family GTPases, membrane tethering complexes and soluble *N*-ethylmaleimide-sensitive factor attachment protein receptor (SNARE) proteins (Barr and Lambright, 2010; Burd and Cullen, 2014; Jahn and Scheller, 2006; Numrich and Ungermann, 2014; Pfeffer, 2017; Stenmark, 2009; van Weering et al., 2010). Guanine nucleotide exchange factors (GEFs) belonging to the conserved VPS9 family are important regulators of endosomal function that activate endosomal Rab5-like GTPases (Carney et al., 2006; Delprato and Lambright, 2007). In yeast, the VPS9-domain GEFs Muk1 and Vps9 stimulate the endosomal Rabs Vps21, Ypt52 and Ypt53 to perform downstream functions including activating phosphoinositide 3-kinase (PI3K) to produce the anionic lipid species phosphatidylinositol 3-phosphate (PI3P; Christoforidis et al., 1999; Hama et al., 1999; Paulsel et al., 2013; Peplowska et al., 2007; Singer-Krüger et al., 1994). Together, PI3P and endosomal Rabs are important determinants of endosomal identity and are responsible for recruiting effectors, including coat proteins and vesicle tethers, to the endosomal membrane.

Sorting nexins (SNXs) are a conserved family of proteins that perform direct roles in endosomal trafficking by binding to transmembrane cargo proteins and enriching them into sorting domains (Carlton et al., 2005; Hong, 2019). SNXs localize to the endosome through conserved Phox homology (PX) domains that typically recognize PI3P (Cheever et al., 2001; Xu et al., 2001). A sub-family of SNXs known as SNX-BARs additionally contain a Bin/Amphiphysin/RVS (BAR) domain that mediates dimerization with other BAR domain-containing proteins and imparts membrane binding/deforming properties (Frost et al., 2009; van Weering et al., 2010; van Weering et al., 2012). These SNX-BAR dimers, which are capable of deforming cargo-rich membranes into sorting tubules, are emerging as important regulators of protein transport. The seven SNX-BAR proteins present in yeast include the conserved retromer subunits Vps5 and Vps17 (Horazdovsky et al., 1997), the SNX8 homolog Mvp1 (Suzuki et al., 2021), the SNX4 homolog Snx4 and its partners Snx41 and Atg20 (SNX7 and SNX30 in humans, respectively; Hettema et al., 2003) and the Vps5 paralog Ykr078w. Retromer and Snx4 complexes promote cargo sorting from the endosome and vacuole (Arlt et al., 2015; Suzuki and Emr, 2018), while Mvp1 appears to function only at the endosome (Suzuki et al., 2021). There is no known sorting function for Ykr078w, which lacks a clear subcellular localization.

The best characterized of these SNX-BAR-containing complexes is the heteropentameric retromer complex, which is composed of the Vps26-Vps35-Vps29 trimer and the Vps5-Vps17 SNX-BAR dimer (Seaman et al., 1998). Retromer localizes to PI3P-rich membranes where it promotes the retrograde sorting of cargo including the well-characterized carboxypeptidase Y (CPY) receptor Vps10 (Burda et al., 2002; Seaman et al., 1998; Seaman et al., 1997). In metazoans, the term retromer refers specifically to the VPS26-VPS35-VPS29 trimer which associates with a variety of adaptor proteins including SNXs to promote cargo sorting (Cullen and Korswagen, 2011; Gallon and Cullen, 2015). Mutations in VPS35 have been linked to neurodegenerative conditions including Parkinson’s disease and Alzheimer’s disease (Mohan and Mellick, 2017; Rahman and Morrison, 2019; Vilariño-Güell et al., 2011; Wen et al., 2011; Zimprich et al., 2011), establishing a connection between endosomal transport machinery and human neurological health.

We previously identified a physical association between yeast retromer and the VPS9-domain GEFs Vps9 and Muk1, whose activity is required to maintain endosomal pools of PI3P for retromer recruitment (Bean et al., 2015). We also identified a novel VPS9-domain protein, Vrl1, which is mutated and non-functional in strains previously used for trafficking studies. Vrl1 can function as the sole VPS9-domain GEF to stimulate production of endosomal PI3P. Notably, the human homolog of Vrl1, VARP, physically associates with retromer to drive an endosome-to-plasma membrane sorting pathway (Hesketh et al., 2014). Here, we show that Vrl1 is a member of the SNX-BAR protein family and that it specifically binds the Vps5 paralog Ykr078w/Vin1 to form what we now call the VINE complex. Our results suggest that VINE is both a VPS9-domain GEF and a SNX-BAR coat complex that may operate alongside the retromer, Mvp1 and Snx4 pathways.

## Results

### Vrl1 is a predicted PX-BAR protein that interacts with conserved machinery at the endosome

**Figure 1.**
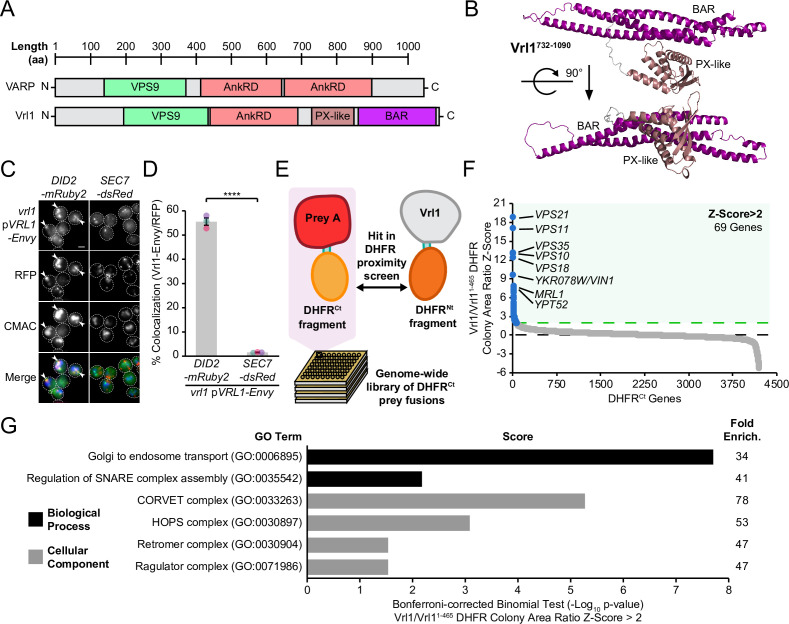
Vrl1 is a predicted PX-BAR protein that interacts with conserved machinery at the endosome. (**A**) Schematic of Vrl1 and VARP domain architecture. (**B**) ColabFold predicts the Vrl1 C-terminus has a SNX-BAR-like PX and BAR domain fold. (**C**) Vrl1-Envy colocalizes with Did2-mRuby2-labeled endosomes, but not with the Sec7-dsRed Golgi marker. (**D**) Quantification of colocalization as the percentage of Vrl1 puncta overlapping RFP puncta in *C*. Two-tailed equal variance *t* test; n=3, cells/strain/replicate ≥1395; ****=p < 0.0001. (**E**) Schematic of DHFR proximity screen methodology. (**F**) Z-score distribution of the ratio of colony areas from genome-wide DHFR screens of full-length and truncated Vrl1 baits that localize to the endosome and cytosol, respectively. (**G**) Gene Ontology (GO) functional enrichment analysis of Vrl1 DHFR interactors (Z-score >2; http://geneontology.org). GO terms of the most specific hierarchical subclass with a fold enrichment value >25 are presented as the negative base 10 log of the associated p-value from a Bonferroni-corrected binomial test of significance. Scale bars, 2 µm. Error bars report standard error of the mean (SEM). Enrich., enrichment. aa, amino acids. Figure 1—source data 1.Data associated with Figure 1D. Figure 1—source data 2.Data associated with Figure 1G.

Vrl1 and its human ortholog VARP share a VPS9 domain, as well as a conserved N-terminus and ankyrin repeat domain (AnkRD) that are not found in other yeast VPS9-domain GEFs (Herman et al., 2018; Figure 1A). Vrl1 also features a ~350 amino acid (aa) unannotated region downstream of the AnkRD that is not present in VARP. The protein fold recognition program Phyre2 (Kelley et al., 2015) identified a PX-BAR module with very high confidence (98.6%) in this region (aa 737–1089; Figure 1—figure supplement 1A), and ab initio modeling of this region by AlphaFold2-powered ColabFold software (Mirdita et al., 2022) predicted a structure with striking similarity to the PX-BAR fold (Figure 1B, Figure 1—figure supplement 1B, C). Because the predicted Vrl1 PX domain is missing key residues for PI3P binding (Figure 1—figure supplement 1D), we refer to it as a ‘PX-like’ domain. To our knowledge, Vrl1 is the first VPS9 domain-containing protein with predicted structural homology to the SNX-BAR family.

**Figure 1—figure supplement 1.**
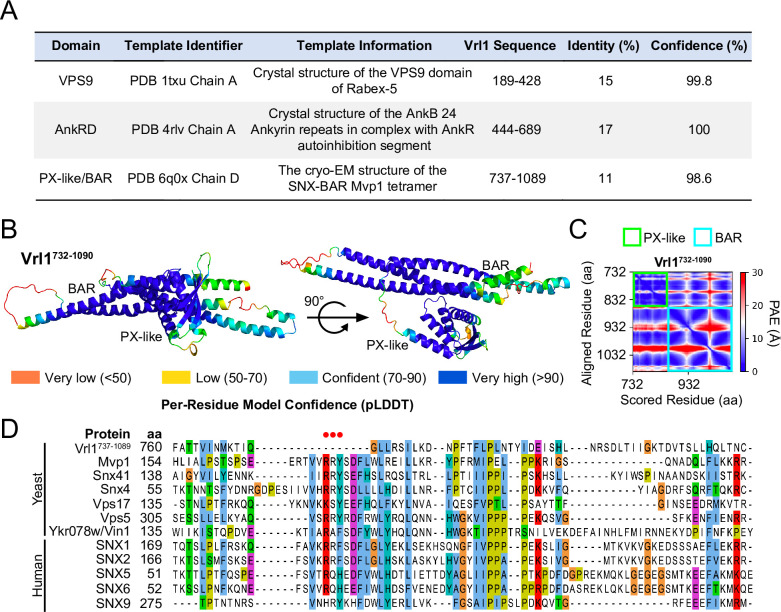
The Vrl1 PX-like domain is missing key PI3P-binding residues. (**A**) Results from Phyre2 analysis of Vrl1 sequences (Intensive mode, http://www.sbg.bio.ic.ac.uk/phyre2). (**B**) ColabFold-predicted Vrl1 C-terminus with the predicted local-difference distance test (pLDDT; Jumper et al., 2021; Mirdita et al., 2022) scores mapped to each residue. (**C**) ColabFold-generated predicted alignment error (PAE; Jumper et al., 2021; Mirdita et al., 2022) plot for the Vrl1 C-terminus demonstrates a high confidence folding prediction. (**D**) Sequence alignment of Vrl1 C-terminus (aa 737–1089) with yeast and human PX domain-containing proteins. The canonical PI3P-binding ‘RRY’ motif is absent in Vrl1 (highlighted with red dots above alignment). aa, amino acids.

**Figure 1—figure supplement 2.**
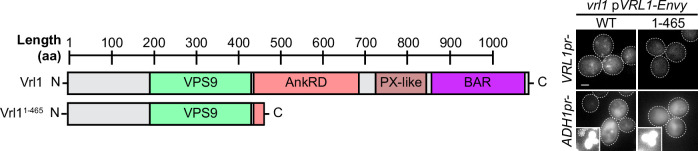
The Vrl1 N-terminus and VPS9 domain are not sufficient to localize to puncta. The Vrl1 N-terminus (aa 1–465) does not localize to puncta when expressed from the endogenous *VRL1* promoter (*VRL1pr*) or the strong *ADH1pr* and instead accumulates in the cytosol. Insets are scaled to match other images in the same channel (see materials and methods for details). Scale bars, 2 µm. WT, wild type.

We found that Vrl1, when C-terminally tagged with the bright GFP variant Envy, was present at perivacuolar puncta that colocalize with the endosomal marker Did2-mRuby2, but not the Golgi protein Sec7-dsRed (56% and 2%, respectively, p<0.0001; Figure 1C, D). These observations indicate that unlike other yeast VPS9-domain GEFs (Paulsel et al., 2013), Vrl1 constitutively localizes to endosomes. To identify endosomal partners of Vrl1, we performed a protein fragment complementation assay (PCA) based on a drug-resistant variant of the dihydrofolate reductase (DHFR) enzyme (Figure 1E; Michnick et al., 2010; Tarassov et al., 2008). Proximity between two proteins that are fused to complementary DHFR fragments reconstitutes enzyme activity and confers resistance to the inhibitor methotrexate. Full-length Vrl1, and a cytosolic fragment of Vrl1 lacking the AnkRD, PX-like and BAR domains (Vrl1^1-465^; Figure 1—figure supplement 2), were expressed as DHFR^Nt^ fusions under the control of the constitutive *ADH1* promoter (*ADH1pr*). Z-scores were generated from the colony area ratio of full-length Vrl1 vs Vrl1^1-465^ (Figure 1F, Supplementary file 1). This identified the endosomal Rab GTPases Vps21 (Z=18.7) and Ypt52 (Z=7.6), and other conserved endosomal proteins including the retromer subunit Vps35 (Z=13), the hydrolase receptors Vps10 (Z=12.9) and Mrl1 (Z=7.7), and components of the Class C Core complex Vps11 (Z=16.9) and Vps18 (Z=12.2; Figure 1F). Functional enrichment analysis of Vrl1 interactors (Z>2) highlighted relationships with other subunits of endosomal complexes including retromer and the CORVET complex (Figure 1G, Supplementary file 2; Ashburner et al., 2000; Gene Ontology Consortium, 2021). These results suggest that Vrl1 is an endosomal SNX-BAR-like protein that contacts both membrane tethering and trafficking machinery.

### Vrl1 and the Vps5 paralog Vin1 form the VINE complex

**Figure 2.**
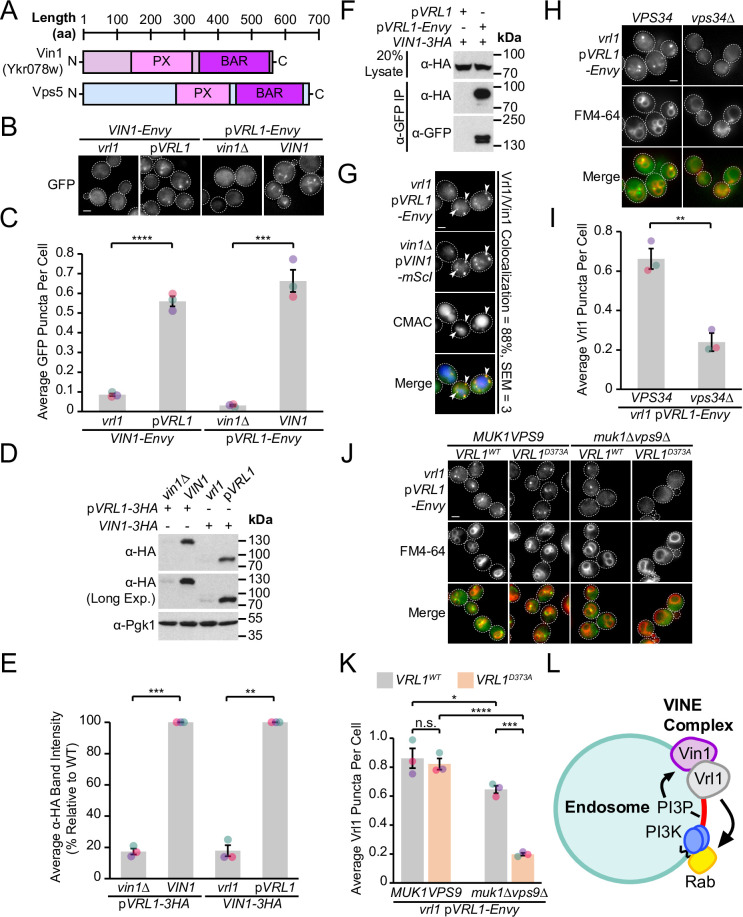
Vrl1 and the Vps5 paralog Vin1 form the VINE complex. (**A**) Schematic of Ykr078w (Vin1) and its paralog Vps5. (**B**) Vin1-Envy and Vrl1-Envy require Vrl1 and Vin1, respectively, for localization to puncta. (**C**) Quantification of Vin1-Envy and Vrl1-Envy puncta per cell in *B*. Two tailed equal variance *t* tests; n=3, cells/strain/replicate ≥1,879; ***=p < 0.001, ****=p < 0.0001. (**D**) Vrl1-3HA and Vin1-3HA require Vin1 and Vrl1, respectively, for protein stability by western blot. Pgk1 serves as a loading control. (**E**) Quantification of Vrl1-3HA and Vin1-3HA levels in *D* by densitometry. Two tailed Welch’s *t* tests; n=3, **=p < 0.01, ***=p < 0.001. (**F**) Co-immunoprecipitation (CoIP) of Vin1-3HA with Vrl1-Envy suggests stable complex formation. (**G**) Vrl1-Envy colocalizes with Vin1-mScI at perivacuolar puncta. (**H**) Vrl1-Envy requires the PI3K catalytic subunit Vps34 for punctate localization. (**I**) Quantification of Vrl1-Envy puncta per cell in *H*. Two-tailed equal variance *t* test; n=3, cells/strain/replicate ≥897; **=p < 0.01. (**J**) Vrl1-Envy localization in the absence of VPS9-domain GEFs is dependent on the Vrl1 catalytic residue D373. (**K**) Quantification of Vrl1-Envy puncta per cell in *J*. One-way ANOVA with Tukey’s multiple comparison test; n=3, cells/strain/replicate ≥1705; not significant, n.s.=p > 0.05, *=p < 0.05, ***=p < 0.001, ****=p < 0.0001. (**L**) Model of the Vin1 and Vrl1-containing VINE complex at endosomes. Scale bars, 2 µm. Error bars report SEM. Exp., Exposure. WT, wild type. Figure 2—source data 1.Data associated with Figure 2C. Figure 2—source data 2.Data associated with Figure 2E. Figure 2—source data 3.Data associated with Figure 2G. Figure 2—source data 4.Data associated with Figure 2I. Figure 2—source data 5.Data associated with Figure 2K. Figure 2—source data 6.Uncropped blot data associated with Figure 2D. Figure 2—source data 7.Uncropped blot data associated with Figure 2F.

Our DHFR screen identified a strong connection between Vrl1 and the uncharacterized SNX-BAR Ykr078w (Z=9.5), the paralog of membrane-binding retromer subunit Vps5 (Byrne and Wolfe, 2005; Horazdovsky et al., 1997), which we have named ‘Vrl1-Interacting Sorting Nexin 1’ or Vin1 (Figure 2A). Vin1 has a reported cytosolic distribution (Huh et al., 2003), which is surprising given that its paralog Vps5 localizes to endosomes in a PI3P-dependent manner (Burda et al., 2002) and that Vin1 interacts with PI3P in vitro (Yu and Lemmon, 2001). Increasing Vin1 levels did not alter its cytosolic distribution pattern which we observed in both endogenously expressed N- and C-terminally tagged strains (Figure 2B; Figure 2—figure supplement 1A). Since common laboratory *S. cerevisiae* strains carry the non-functional mutant *vrl1* allele (Bean et al., 2015), we wondered if complementing this mutation with a plasmid-expressed copy of *VRL1* featuring the corrected sequence (p*VRL1*) would affect the localization of Vin1. Indeed, we found that expression of *VRL1* caused a dramatic redistribution of Vin1-Envy from the cytosol to intracellular puncta (p<0.0001; Figure 2B, C; Figure 2—figure supplement 1A), and that over-expressing *VRL1* from the *ADH1pr* further increased the number of bright Vin1-Envy puncta (p<0.0001; Figure 2—figure supplement 1B, C). Deletion of *VIN1* prevented Vrl1-Envy from forming intracellular puncta (p<0.001; Figure 2B, C), suggesting that the localization of Vrl1 and Vin1 is highly interdependent.

**Figure 2—figure supplement 1.**
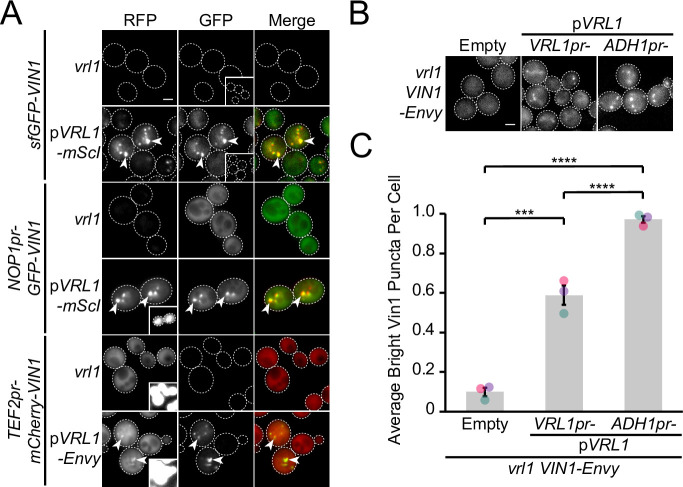
Vrl1 is indispensable for Vin1 puncta localization. (**A**) N-terminally tagged Vin1 requires Vrl1 for localization to puncta at three different expression levels. By relative fluorescence, the *NOP1* promoter (*NOP1pr*) is stronger than the *VIN1pr* and the *TEF2pr* is stronger than the *NOP1pr*. Insets are scaled to match other images in the same channel (see Materials and methods for details). (**B**) Over-expression of Vrl1 from the *ADH1pr* increases the average number of bright Vin1-Envy puncta per cell. (**C**) Quantification of Vrl1-Envy puncta per cell in *B*. One-way ANOVA with Tukey’s multiple comparison test; n=3, cells/strain/replicate ≥1495; ***=p < 0.001, ****=p < 0.0001. Scale bars, 2 µm. Error bars report SEM. Figure 2—figure supplement 1—source data 1.Data associated with Figure 2—figure supplement 1C.

Vrl1 and Vin1 are also dependent on each other for stability, as the levels of triple hemagglutinin (3HA)-tagged Vrl1 and Vin1 were severely reduced in strains lacking *VIN1* or *VRL1* (17% of WT, p<0.001 and 18% of WT, p<0.01, respectively; Figure 2D, E). We found that Vin1-3HA strongly co-purified with Vrl1-Envy (64% recovery of Vin1; Figure 2F), and Vrl1-Envy and Vin1-mScarletI (-mScI) showed a high degree of colocalization at endosomal puncta (88%; Figure 2G), suggesting that these proteins form a complex.

Since both Vin1 and Vrl1 have predicted PX domains, we wondered if, like other SNX-BARs, they bind PI3P at endosomes. Vrl1 was displaced to the cytosol in a PI3K deletion mutant (*vps34*Δ, p<0.01; Figure 2H, I). Since the Vrl1 PX domain is missing residues that are typically required to bind PI3P, this suggests the PX domain of Vin1 is important for endosomal recruitment. Indeed, a recent study demonstrated that Vin1, therein referred to as Vps501, binds to PI3P through an unconventional motif in its PX domain (Goyal et al., 2022). PI3K is activated by Rab5-like GTPases (Christoforidis et al., 1999), which in turn require VPS9-domain GEFs for their activity (Carney et al., 2006; Delprato and Lambright, 2007). We found that, in a *muk1*Δ*vps9*Δ strain that lacks all other VPS9-domain GEFs, Vrl1 localization is dependent on a conserved catalytic residue in the VPS9 domain (Vrl1^D373^; Bean et al., 2015; p<0.001; Figure 2J, K), suggesting that Vrl1 may leverage its ability to stimulate endosomal PI3P production and promote its own membrane recruitment.

Taken together, our results suggest that Vrl1 and Vin1 form a novel complex that localizes to endosomes in a PI3P-dependent manner (Figure 2L). Since neither Vrl1 nor Vin1 is stable or capable of membrane localization in the absence of the other, we reason that these proteins primarily exist as members of this complex which we have named the ‘VPS9 GEF-Interacting Sorting Nexin’ or VINE complex.

### Vrl1 is predicted to form a BAR-BAR dimer with both Vin1 and Vps5

**Figure 3.**
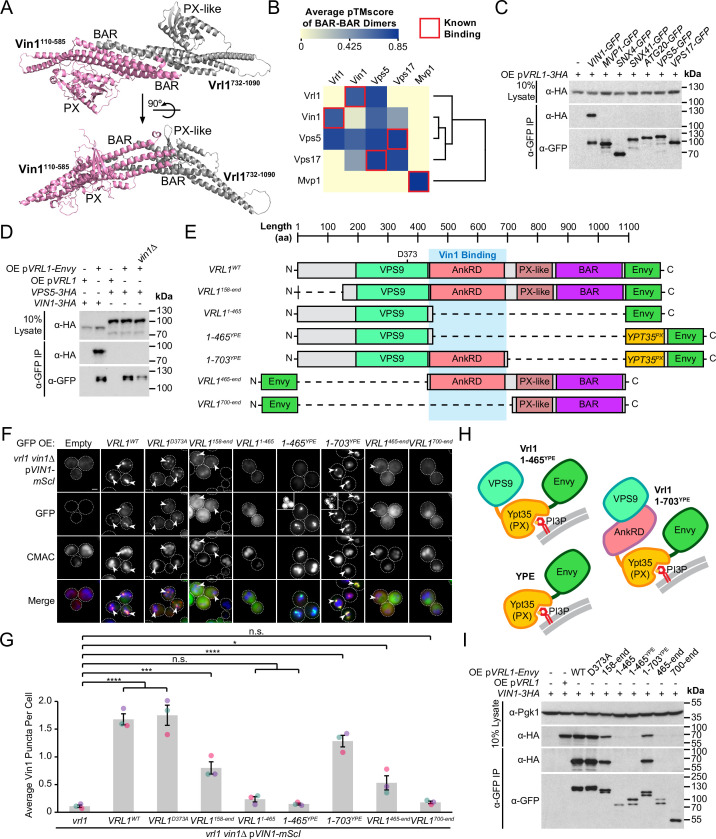
Vrl1 interacts with Vin1 primarily through the AnkRD. (**A**) ColabFold-predicted physical interaction of Vrl1 and Vin1 BAR domains along the canonical BAR-BAR dimerization interface. pTMscore = 0.75. (**B**) Matrix of ColabFold-predicted BAR-BAR dimers for select yeast SNX-BARs. Hierarchical clustering was performed using an uncentered Pearson correlation with average linkage. (**C**) Vin1 is the only yeast SNX-BAR that interacts with over-expressed Vrl1-3HA by CoIP. (**D**) Vps5-3HA does not bind to Vrl1-Envy in a strain lacking Vin1. (**E**) Schematic of Envy-tagged Vrl1 truncations and chimeras in *F*. (**F**) The Vrl1 AnkRD is necessary to recruit Vin1-mScI to puncta. Images with very bright signals use custom settings to show protein localization; insets are scaled identically to other images in the same channel (see materials and methods for details). (**G**) Quantification of Vin1-mScI puncta per cell in *F*. One-way ANOVA with Dunnett’s multiple comparison test; n=3, cells/strain/replicate ≥764; not significant, n.s.=p > 0.05, *=p < 0.05, **=p < 0.01, ***=p < 0.001, ****=p < 0.0001. (**H**) Diagram of chimeric Vrl1 fusion proteins that are artificially recruited to the endosomal system by the PX domain of sorting nexin Ypt35. (**I**) The Vrl1 AnkRD is necessary for physical interaction with Vin1-3HA by CoIP. Pgk1 serves as a loading control. Scale bars, 2 µm. Error bars report SEM. OE, over-expressed. YPE, Ypt35(PX)-Envy. WT, wild type. Figure 3—source data 1.Data associated with Figure 3B. Figure 3—source data 2.Data associated with Figure 3G. Figure 3—source data 3.Uncropped blot data associated with Figure 3C. Figure 3—source data 4.Uncropped blot data associated with Figure 3D. Figure 3—source data 5.Uncropped blot data associated with Figure 3I.

SNX-BAR proteins interact via an extensive hydrophobic interface between the BAR domains (van Weering et al., 2012). ColabFold software (Mirdita et al., 2022) predicted that the PX-like and BAR domains of Vrl1 (aa 732–1090) bind to the PX and BAR domains of Vin1 (aa 110–585) to form a canonical SNX-BAR dimer (pTMscore = 0.75; Figure 3A, Figure 3—figure supplement 1A). To assess the accuracy of ColabFold in predicting specific BAR domain pairings, we systematically modeled pairwise homotypic and heterotypic interactions of all yeast SNX-BAR proteins (Figure 3B, Supplementary file 3). This accurately predicted the homodimerization of Mvp1 (Suzuki et al., 2021) and the heterodimerization of Vps5/Vps17 (Seaman and Williams, 2002). Neither Vrl1 nor Vin1 was predicted to form homodimers, although unexpectedly Vrl1 was predicted to pair equally well with both Vin1 and its paralog Vps5. By comparing plots of predicted alignment error (PAE) for different combinations of SNX-BARs, we found that Vrl1 and Vps5 exhibit high confidence interactions with Vin1 and Vps17, respectively, whereas Vrl1 and Vps17 were predicted not to interact (Figure 3—figure supplement 1B).

**Figure 3—figure supplement 1.**
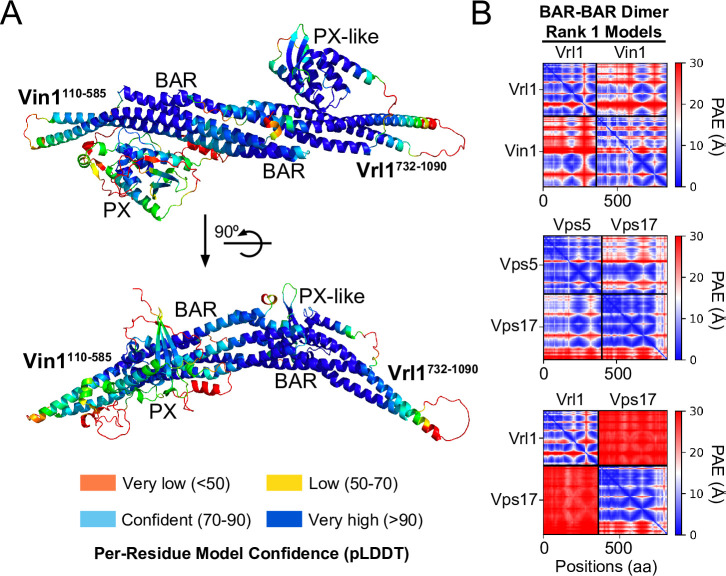
Confidence measures of yeast SNX-BAR dimer predictions. (**A**) ColabFold-predicted physical interaction of Vrl1 and Vin1 BAR domains with the predicted local-difference distance test (pLDDT; Jumper et al., 2021; Mirdita et al., 2022) scores mapped to each residue. (**B**) ColabFold-generated predicted alignment error (PAE; Jumper et al., 2021; Mirdita et al., 2022) plots for yeast SNX-BAR pairs demonstrate confidence of inter-chain contacts, indicating the relative likelihood of functional pairings. On-diagonal boxes with low PAE values reflect confidently predicted intramolecular interactions while off-diagonal boxes with low PAE values reflect confidently predicted intermolecular interactions between two separate polypeptide sequences. aa, amino acids.

**Figure 3—figure supplement 2.**
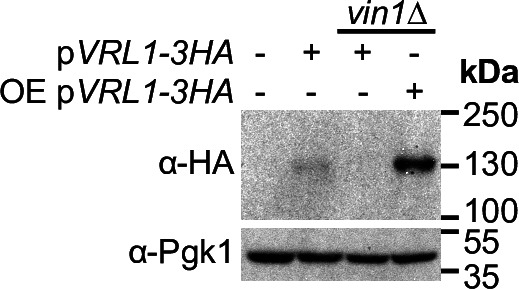
Over-expression of *VRL1* restores protein levels in a *vin1*Δ mutant. Vrl1 protein levels are restored in a *vin1*Δ mutant by western blot when *VRL1* is over-expressed on the *ADH1pr*. Pgk1 serves as a loading control. Figure 3—figure supplement 2—source data 1.Uncropped blot data associated with Figure 3—figure supplement 2.

**Figure 3—figure supplement 3.**
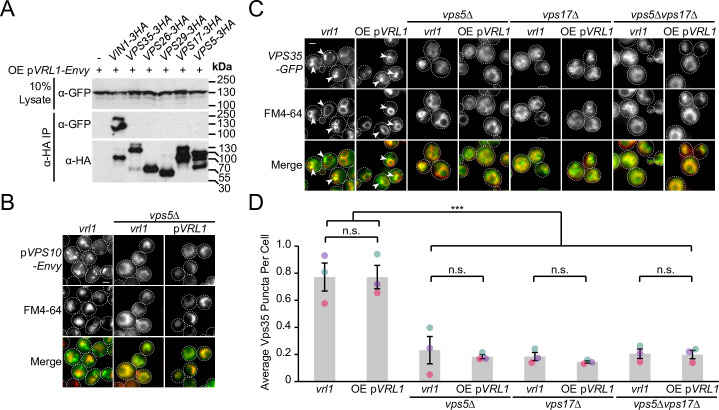
Vrl1 does not form a novel retromer-like complex. (**A**) Vrl1 does not interact strongly with subunits of retromer by CoIP. (**B**) Expression of *VRL1* does not rescue loss of endosomal Vps10-Envy in a *vps5*∆ strain. (**C**) Expression of *VRL1* does not rescue loss of endosomal Vps35-GFP or vacuolar morphology defects in retromer SNX-BAR deletion strains. (**D**) Quantification of Vps35-GFP puncta per cell in *C*. One-way ANOVA with Tukey’s multiple comparison test; n=3, cells/strain/replicate ≥1243; not significant, n.s.=p > 0.05, ***=p < 0.001. Scale bars, 2 µm. Error bars report SEM. OE, over-expressed. Figure 3—figure supplement 3—source data 1.Data associated with Figure 3—figure supplement 3D. Figure 3—figure supplement 3—source data 2.Uncropped blot data associated with Figure 3—figure supplement 3A.

Given that Vrl1 was predicted to interact with both Vin1 and Vps5, we wondered if Vrl1 could functionally partner with Vps5 to form a novel retromer-like complex. First, we tested all known yeast SNX-BAR proteins for their ability to bind Vrl1 and found that Vin1 alone interacts with Vrl1 (Figure 3C). Vrl1 also failed to bind Vps5 when *VIN1* was deleted from a strain that over-expresses Vrl1 to compensate for its instability in the *vin1*∆ mutant (Figure 3D; Figure 3—figure supplement 2), suggesting that a possible Vrl1/Vps5 interaction was not overlooked due to competition from Vin1. Further, Vrl1 did not interact with any of the other retromer subunits (Figure 3—figure supplement 3A), but this assay could fail to detect weak or transient interactions. Using functional readouts, we found that Vrl1 was unable to promote the endosomal localization of Vps10 (Figure 3—figure supplement 3B) or Vps35 (Figure 3—figure supplement 3C, D) in strains lacking Vps5 and/or Vps17, indicating that Vrl1 does not functionally pair with retromer SNX-BARs and that Vin1/Vrl1 cannot replace Vps5/17 to form a retromer-like complex. These results further suggest that Vrl1 has strong paralog specificity and that interactions beyond the BAR-BAR interface could be responsible for its specific recognition of Vin1.

### Vrl1 interacts with Vin1 primarily via the AnkRD

To identify regions critical for Vrl1/Vin1 binding, we quantified the membrane recruitment of Vin1-mScarletI in cells expressing a series of Envy-tagged Vrl1 fragments (Figure 3E, F). We found that the GEF-deficient mutant (Vrl1^D373A^; Bean et al., 2015) and the N-terminal truncation (Vrl1^158-end^) significantly recruited Vin1 (p<0.0001 and p<0.001, respectively; Figure 3G) despite the weaker punctate localization of the Vrl1^158-end^ construct relative to WT, which could explain its reduced recruitment of Vin1. Deletion of C-terminal sequences (i.e. Vrl1^1-465^) blocked the membrane localization of both Vrl1 and Vin1. To test the role of the Vrl1 PX-like and BAR domains, we replaced this region with a localization module consisting of the PI3P-binding PX domain of Ypt35 fused to Envy which we refer to as ‘YPE’ (Figure 3H). Strikingly, the resulting Vrl1(1-703)^YPE^ chimera strongly recruited Vin1 to puncta (*P*<0.0001; Figure 3G), suggesting that BAR-BAR interactions are dispensable for Vin1 recruitment. A further truncation that removed the AnkRD to create Vrl1(1-465)^YPE^ localized to endosomes yet failed to recruit Vin1 (Figure 3F, G), indicating that the AnkRD contains a potent interacting interface for Vin1. In support of this idea, the Vrl1^465-end^ fragment which contains the AnkRD, PX-like and BAR domains weakly localized and recruited a small but significant amount of Vin1 (p<0.05) while the Vrl1^700-end^ fragment containing only the PX-like and BAR domains did not localize or recruit Vin1 (Figure 3F,G).

We then tested the Vrl1 truncation series (Figure 3E) for the ability to CoIP Vin1-3HA (Figure 3I), and found the Vrl1 constructs that strongly recruited Vin1 to puncta also showed physical interactions by CoIP. Taken together, the Vin1 recruitment assays, CoIPs and structural predictions suggest that the VINE complex assembles primarily through an interaction between Vin1 and the Vrl1 AnkRD, while a secondary interaction between the Vin1 and Vrl1 BAR domains may occur at the endosomal membrane.

### The Vrl1 AnkRD recognizes a small region of the disordered Vin1 N-terminus

**Figure 4.**
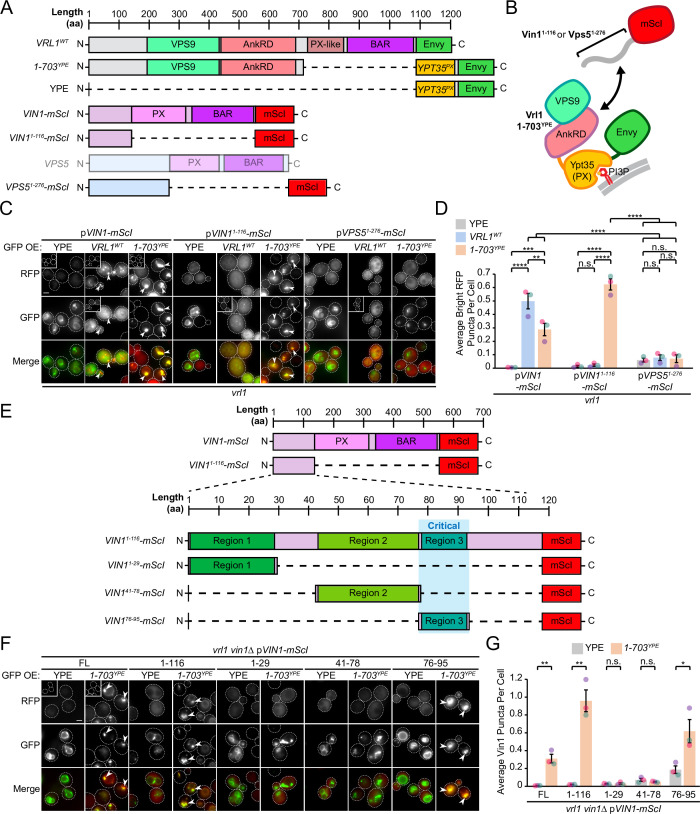
The Vrl1 AnkRD recognizes a small region of the Vin1 N-terminus. (**A**) Schematic of constructs used in *C, D*. Full-length Vps5 was not tested but is shown for comparison. (**B**) Diagram of chimeric Vrl1 recruitment assay used to test for interactions with the unstructured N-terminus of either Vps5 (Vps5^1-276^) or Vin1 (Vin1^1-116)^. (**C**) The AnkRD-containing Vrl1(1-703)^YPE^ chimera recruits the N-terminus of Vin1, but not Vps5. Insets are scaled to match other images in the same channel (see Materials and methods for details). (**D**) Quantification of RFP puncta per cell in *C*. One-way ANOVA with Tukey’s multiple comparison test; n=3, cells/strain/replicate ≥902; not significant, n.s.=p > 0.05, **=p < 0.01, ***=p < 0.001, ****=p < 0.0001. (**E**) Schematic of Vin1 N-terminal fragments used to map the Vrl1 recruitment site. (**F**) The AnkRD-containing Vrl1(1-703)^YPE^ chimera recruits a small fragment of the Vin1 N-terminus. Insets are scaled to match other images in the same channel. (**G**) Quantification of Vin1-mScI puncta per cell in *F*. Two-tailed equal variance *t* tests; n=3, cells/strain/replicate ≥294; not significant, n.s.=p > 0.05, *=p < 0.05, **=p < 0.01. Scale bars, 2 µm. Error bars report SEM. OE, over-expressed. FL, full-length. WT, wild type. YPE, Ypt35(PX)-Envy. Figure 4—source data 1.Data associated with Figure 4D. Figure 4—source data 2.Data associated with Figure 4G.

AnkRD interactions may explain how Vrl1 discriminates between Vin1 and its paralog Vps5. Vin1 and Vps5 have unstructured N-terminal regions preceding their respective PX domains (Vin1^1-116^ and Vps5^1-276^; Figure 4A, Figure 4—figure supplement 1). When we expressed the N-terminal regions of Vin1 or Vps5 fused to mScarletI (Figure 4B, C), we observed strong recruitment of the Vin1 N-terminus by the Vrl1(1-703)^YPE^ chimera, but not the YPE module alone (p<0.0001; Figure 4D). In contrast, we detected no recruitment of the Vps5 N-terminus by any of our tested constructs suggesting that the N-terminal regions of the paralogous SNX-BARs dictate specificity for Vrl1.

**Figure 4—figure supplement 1.**
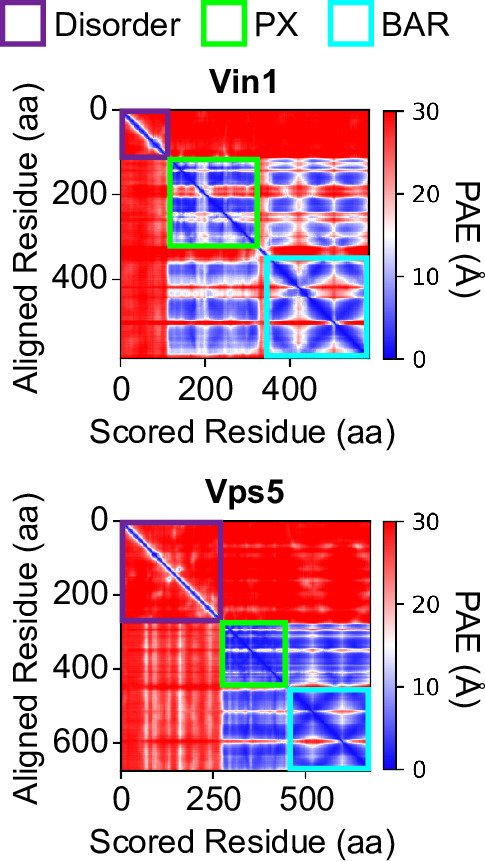
The N-terminal regions of Vin1 and Vps5 are predicted to be disordered. A lack of off-diagonal signal in the ColabFold-generated predicted alignment error (PAE; Jumper et al., 2021; Mirdita et al., 2022) plots of Vin1 and Vps5 indicates a shared lack of structure in the N-terminus of either protein. On-diagonal signal for the PX and BAR domains provides a point of comparison for how structured domains appear in prediction PAE plots. aa, amino acids.

**Figure 4—figure supplement 2.**
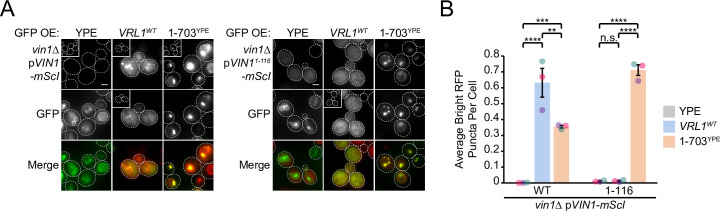
The Vin1 PX-BAR region is indispensable for Vrl1 localization. (**A**) The Vin1 N-terminus is not sufficient to localize over-expressed Vrl1-Envy to membranes in a *VIN1* deletion strain. The Vrl1(1-703)^YPE^ chimera recruits the Vin1 N-terminal prey construct to puncta in strains lacking the chromosomal copy of *VIN1*. Insets are scaled to match other images in the same channel (see Materials and methods for details). (**B**) Quantification of RFP puncta per cell in *A*. One-way ANOVA with Tukey’s multiple comparison test; n=3, cells/strain/replicate ≥1068; not significant, n.s.=p > 0.05, **=p < 0.01, ***=p < 0.001, ****=p < 0.0001. Scale bars, 2 µm. Error bars report SEM. WT, wild type. YPE, Ypt35(PX)-Envy. Figure 4—figure supplement 2—source data 1.Data associated with Figure 4—figure supplement 2B.

**Figure 4—figure supplement 3.**
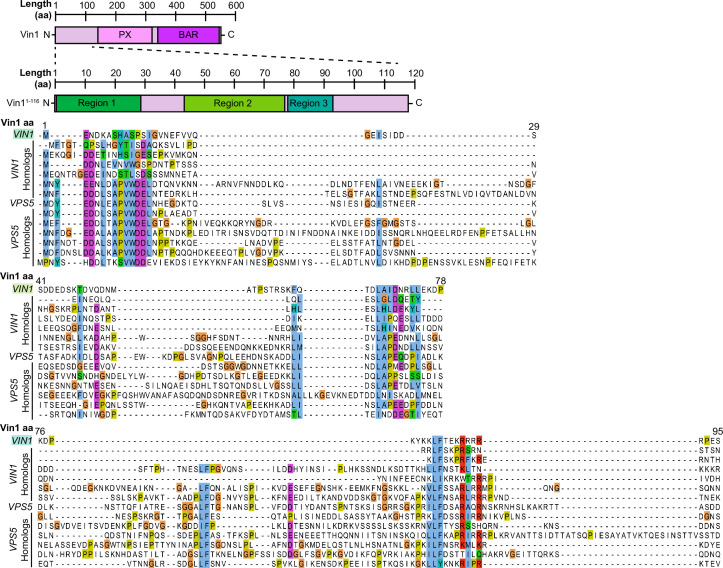
The Vin1 N-terminus has three conserved regions in fungal homologs. Sequence alignment of Vin1 and Vps5 orthologs collected using the Yeast Gene Order Browser (http://ygob.ucd.ie/). Three conserved regions were selected for expression as mScI-tagged fragments: Vin1^1-29^ (Region 1), Vin1^41-78^ (Region 2), and Vin1^76-95^ (Region 3). aa, amino acids.

We noticed that wild type (WT) Vrl1-Envy recruited WT Vin1-mScI to colocalizing puncta (Figure 4C), but was unable to recruit the Vin1 N-terminus. The endogenous, untagged Vin1 may outcompete the Vin1 N-terminal fragment for recruitment by Vrl1, however this could not be tested directly because WT Vrl1 failed to localize when the Vin1 N-terminus was expressed in a *vin1*∆ strain (Figure 4—figure supplement 2A, B). This observation indicates that the Vin1 PX and BAR domains also contribute to VINE assembly and membrane recruitment.

We generated an alignment from fungal orthologs of Vps5 and Vin1 (Figure 4—figure supplement 3; Byrne and Wolfe, 2005) and identified three relatively conserved regions in the Vin1 N-terminus (Figure 4E). When each of these fragments was fused to mScarletI (Figure 4F), only region 3 (Vin1 aa 76–95) was recruited to puncta by Vrl1(1-703)^YPE^ in a *vin1*Δ strain (p<0.05; Figure 4G). This suggests that the Vrl1 AnkRD distinguishes Vin1 from Vps5 through a short sequence in the unstructured Vin1 N-terminus.

**Figure 5.**
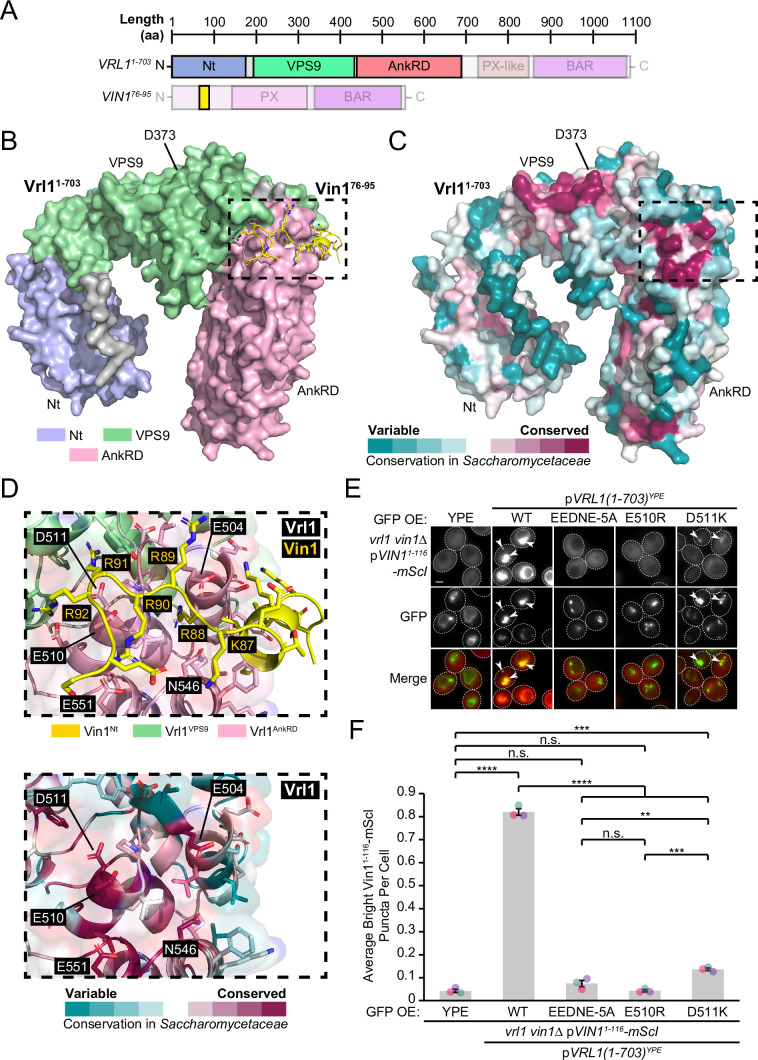
The Vrl1 AnkRD associates with the Vin1 N-terminus through electrostatic interactions. (**A**) Schematic of query sequences used to predict the interact between Vrl1 and the Vin1 N-terminus. Modelled regions are shown as completely opaque. (**B**) ColabFold-predicted interaction between the Vrl1 AnkRD and a minimal fragment of the Vin1 N-terminus (Vin1^76-95^; pTMscore = 0.73). (**C**) Vrl1 sequence conservation within family *Saccharomycetaceae* determined by ConSurf and mapped to a surface model that was predicted by ColabFold. Strong sequence conservation can be seen at the predicted Vin1^76-95^ interacting site and near the catalytic D373 residue. (**D**) *Top:* Vin1^76-95^ is predicted to associate with Vrl1 through a run of basic residues. *Bottom:* Acidic and polar residues in the predicted Vin1-associating Vrl1 AnkRD site are among the most conserved within family *Saccharomycetaceae*. (**E**) Mutation of acidic and polar residues in the Vrl1 AnkRD reduces recruitment of the Vin1 N-terminus by the Vrl1(1-703)^YPE^ chimera. (**F**) Quantification of Vin1^1-116^-mScI puncta per cell in *E*. One-way ANOVA with Tukey’s multiple comparison test; n=3, cells/strain/replicate ≥863; not significant, n.s.=p > 0.05, *=p < 0.05, ***=p < 0.001, ****=p < 0.0001. Scale bars, 2 µm. Error bars report SEM. OE, over-expressed. Nt, N-terminus. WT, wild type. aa, amino acids. YPE, Ypt35(PX)-Envy. Figure 5—source data 1.Data associated with Figure 5F.

ColabFold confidently predicted an interaction between Vrl1(1-703) and the minimal Vin1 fragment (Vin1^76-95^; Figure 5A, B, Figure 5—figure supplement 1A). In this model, Vin1^76-95^ binds Vrl1 at an interface between the VPS9 domain and AnkRD that is conserved in the *Saccharomycetaceae* family (Figure 5C). To identify other potential Vrl1-binding regions, we performed a prediction with the entire Vin1 N-terminal sequence and found that the exact Vin1^76-95^ region that we identified in our subcellular recruitment assay (Figure 4F, G) was the only sequence predicted to associate with Vrl1 (Figure 5—figure supplement 1B, C). The Vin1^76-95^ fragment contains a run of consecutive basic residues (Figure 5D). Interestingly, acidic and polar residues in the corresponding Vrl1 AnkRD interface were among the most conserved within *Saccharomycetaceae* (Figure 5D).

**Figure 5—figure supplement 1.**
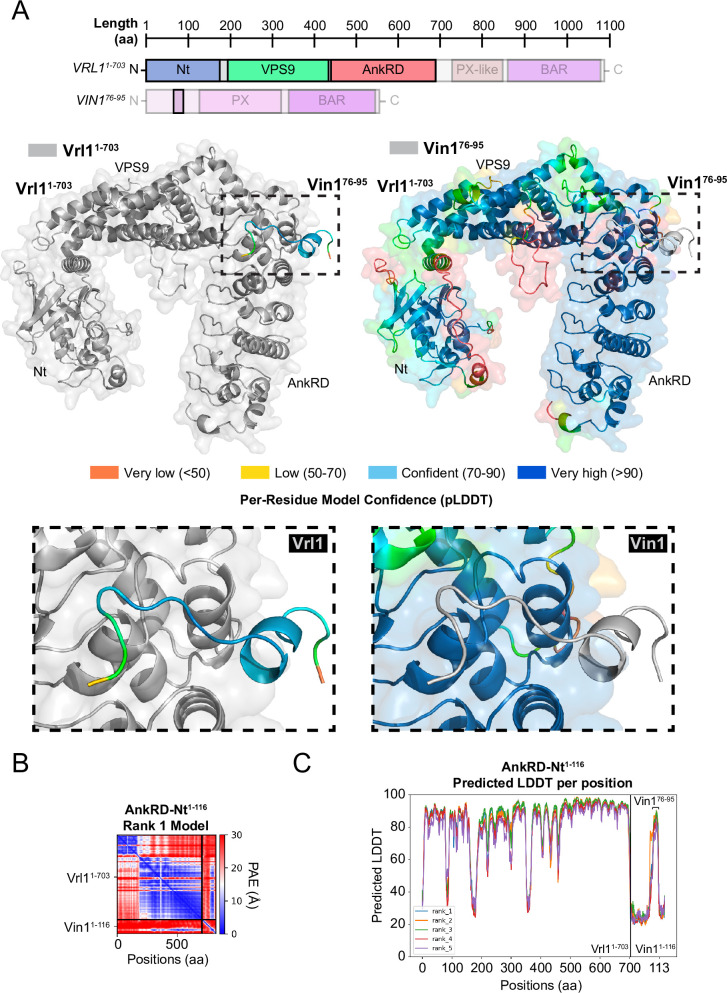
Confidence measures of Vrl1 AnkRD-Vin1 N-terminus binding predictions. (**A**) ColabFold-predicted interaction between the Vrl1 AnkRD and a minimal fragment of the Vin1 N-terminus (Vin1^76-95^; pTMscore = 0.73) with pLDDT scores mapped to each residue of Vin1 (left) or Vrl1 (right). In each case, the interacting protein partner is shown in gray. (**B**) ColabFold-generated predicted alignment error (PAE; Jumper et al., 2021; Mirdita et al., 2022) plot for Vrl1(1-703) and the Vin1 N-terminus (Vin1^1-116^) indicates an intermolecular interaction in the off-diagonal boxes. (**C**) A plot of predicted local-difference distance test (pLDDT; Jumper et al., 2021; Mirdita et al., 2022) scores for five different models of Vrl1(1-703) and Vin1^1-116^ indicates that the minimal region of Vin1 that was recruited by the Vrl1(1-703)^YPE^ chimera, Vin1^76-95^, is the sole confidently predicted region. aa, amino acids. Nt, N-terminus.

**Figure 5—figure supplement 2.**
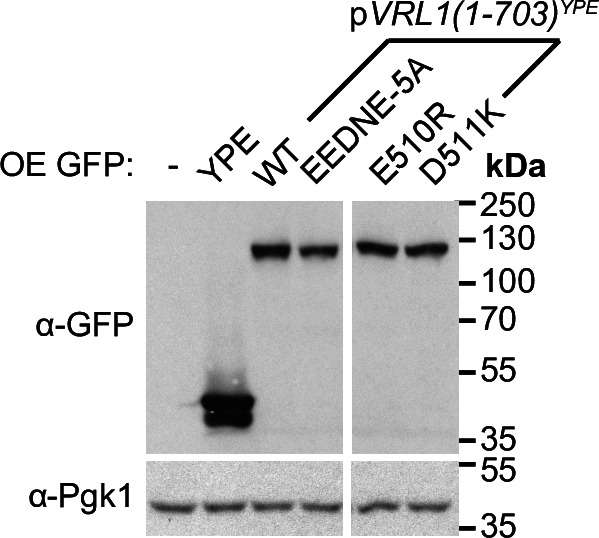
Vrl1(1-703)^YPE^ chimeras with AnkRD mutations are stably expressed. AnkRD-containing Vrl1(1-703)^YPE^ chimeras are stable by western blot when mutations are introduced to sites that are predicted to interact with the Vin1 N-terminus. Pgk1 serves as a loading control. OE, over-expressed. WT, wild type. YPE, Ypt35(PX)-Envy. Figure 5—figure supplement 2—source data 1.Uncropped blot data associated with Figure 5—figure supplement 2.

To assess the relative contribution of residues in the Vrl1 AnkRD site, we generated a series of stable Vrl1 mutants in the context of the Vrl1(1-703)^YPE^ chimera (Figure 5—figure supplement 2). When five of the conserved acidic and polar residues were simultaneously substituted with alanine (EEDNE-5A), recruitment of the Vin1 N-terminus was lost (p<0.0001; Figure 5E, F). In addition, swapping the charges of either E510 or D511 resulted in either complete or severe loss of recruitment, respectively (p<0.0001; Figure 5E, F). These experiments validate the predicted interaction interface in the Vrl1 AnkRD and suggests that Vrl1 binds the Vin1 N-terminus through electrostatic interactions.

### Vin1 regulates Vrl1 GEF activity via membrane localization

**Figure 6.**
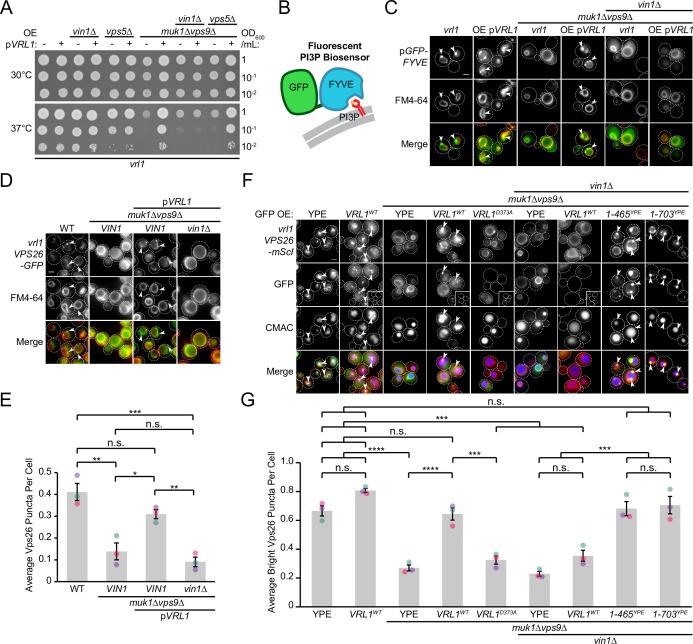
Vin1 controls Vrl1 GEF activity via membrane localization. (**A**) Deletion of *VIN1*, but not *VPS5*, prevents Vrl1 from rescuing the temperature sensitivity of a strain lacking other VPS9-domain GEFs. (**B**) Schematic of PI3P-binding fluorescent biosensor. (**C**) Deletion of *VIN1* prevents Vrl1 from stimulating endosomal PI3P production in a strain lacking other VPS9-domain GEFs. (**D**) Deletion of *VIN1* prevents Vrl1 from rescuing Vps26-GFP localization in a strain lacking other VPS9-domain GEFs. (**E**) Quantification of Vps26-GFP puncta per cell in *D*. One-way ANOVA with Tukey’s multiple comparison test; n=3, cells/strain/replicate ≥1503; not significant, n.s.=p > 0.05, *=p < 0.05, **=p < 0.01, ***=p < 0.001. (**F**) Vin1 is dispensable for Vrl1 activity when fragments containing the N-terminus and VPS9 domain are artificially recruited by a YPE endosomal anchor. Insets are scaled to match other images in the same channel (see materials and methods for details). (**G**) Quantification of Vps26-mScI puncta per cell in *F*. One-way ANOVA with Tukey’s multiple comparison test; n=3, cells/strain/replicate ≥750; not significant, n.s.=p > 0.05, ***=p < 0.001, ****=p < 0.0001. Scale bars, 2 µm. Error bars report SEM. OE, over-expressed. WT, wild type. YPE, Ypt35(PX)-Envy. Figure 6—source data 1.Data associated with Figure 6E. Figure 6—source data 2.Data associated with Figure 6G.

Disruption of the other VPS9-domain GEF proteins results in a severe temperature sensitivity phenotype and loss of endosomal PI3P (Paulsel et al., 2013; Singer-Krüger et al., 1994) that is rescued by Vrl1 in an activity-dependent manner (Bean et al., 2015). We found that deletion of *VIN1,* but not *VPS5*, prevented Vrl1 from rescuing the temperature sensitivity of the *muk1*∆*vps9*∆ strain (Figure 6A). We also found that localization of a fluorescent PI3P biosensor (Figure 6B) was restricted to the vacuolar membrane in *muk1*∆*vps9*∆*vin1*∆ cells expressing Vrl1 (Figure 6C), suggesting that VINE promotes the synthesis of endosomal PI3P only when fully assembled.

We previously found that Vrl1 recovers the PI3P-dependent endosomal localization of retromer in a *muk1*Δ*vps9*Δ strain (Bean et al., 2015). By quantifying the localization of the endogenously tagged retromer subunit Vps26-GFP (Figure 6D), we reproduced this finding and found that deletion of *VIN1* blocked rescue (p<0.01; Figure 6E). We next tested if Vin1 was still required for Vrl1 activity when the PX-like and BAR domains of Vrl1 were replaced by the YPE endosomal anchor (Figure 3H) using Vps26-mScarletI localization as a readout (Figure 6F). We observed that Vrl1(1-465)^YPE^ and Vrl1(1-703)^YPE^, but not WT Vrl1, fully rescued the endosomal localization of Vps26-mScarletI in a *muk1*Δ*vps9*Δ*vin1*∆ strain (p<0.001; Figure 6G). These results suggest that Vin1 regulates the activity of Vrl1 by promoting its localization to endosomes.

### The VINE complex exhibits characteristics of a SNX-BAR coat complex

**Figure 7.**
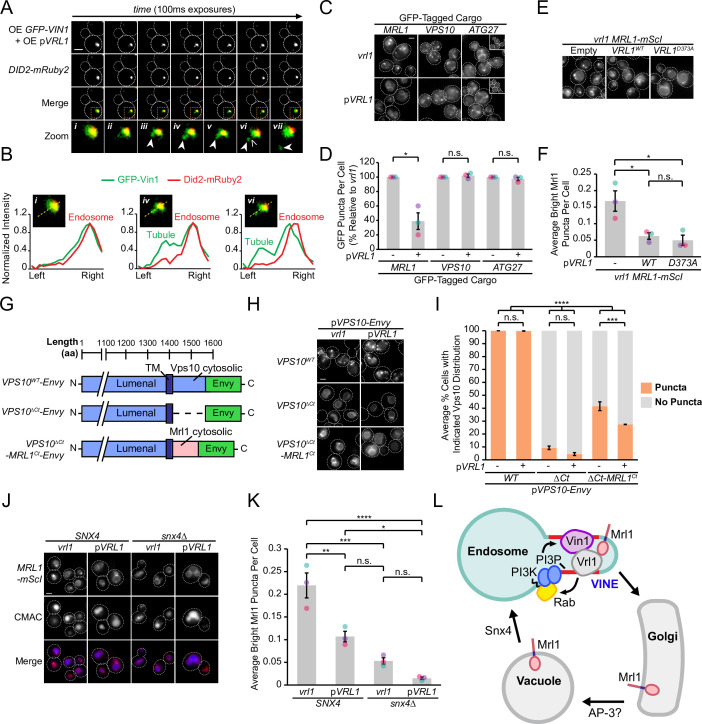
The VINE complex exhibits characteristics of a membrane sorting complex. (**A**) Time-lapse imaging of cells over-expressing GFP-Vin1 and Vrl1 show tubules emanating from Did2-labeled endosomes. Images were uniformly enlarged using a bicubic expansion function to show detail. Solid arrowheads mark a tubule, open arrowhead marks a scission event. (**B**) Normalized intensity line scan analysis performed on images from *A* along the yellow dotted line. (**C**) Punctate localization of GFP-tagged Mrl1, but not other endosomal recycling cargo, is decreased in cells expressing *VRL1*. (**D**) Quantification of GFP-tagged puncta in WT and *vrl1* strains in *C*. Two tailed Welch’s *t* tests; n=3, cells/strain/replicate ≥902; not significant, n.s.=p > 0.05, *=p < 0.05. (**E**) Mutation of the D373 residue required for VPS9 GEF activity does not prevent Vrl1 from redistributing Mrl1. (**F**) Quantification of Mrl1-mScI puncta per cell in *E*. One-way ANOVA with Tukey’s multiple comparison test; n=3, cells/strain/replicate ≥1788; not significant, n.s.=p > 0.05, *=p < 0.05. (**G**) Schematic of Vps10 cytosolic tail mutant and Mrl1 cytosolic tail chimera tested for VINE-mediated sorting in *H, I*. (**H**) The Mrl1 cytosolic tail is sufficient to confer VINE-mediated redistribution. (**I**) Percent of cells showing punctate localization of indicated GFP-tagged constructs in *H*. Blind scoring of GFP signal was conducted manually. One-way ANOVA with Tukey’s multiple comparison test; n=3, cells/strain/replicate ≥237; not significant, n.s.=p > 0.05, ***=p < 0.001, ****=p < 0.0001. (**J**) Mrl1-mScI puncta are reduced in a *snx4*∆ strain. (**K**) Quantification of Mrl1-mScI puncta per cell in *J*. One-way ANOVA with Tukey’s multiple comparison test; n=3, cells/strain/replicate ≥1036; not significant, n.s.=p > 0.05, *=p < 0.05, **=p < 0.01, ***=p < 0.001, ****=p < 0.0001. (**L**) Model for VINE activity and redistribution of Mrl1. VINE promotes its own recruitment to endosomes through a positive feedback loop involving Vrl1 GEF activity and local PI3P production. VINE-coated tubules then recycle cargo, such as Mrl1, from endosomes. VINE may target Mrl1 to the Golgi for subsequent delivery to the vacuolar membrane by the AP-3 complex. Mrl1 is then returned to the endosome by Snx4-containing complexes. See text for details. Scale bars, 2 µm. Error bars report SEM. OE, over-expressed. TM, transmembrane. WT, wild type. Figure 7—source data 1.Data associated with Figure 7B. Figure 7—source data 2.Data associated with Figure 7D. Figure 7—source data 3.Data associated with Figure 7F. Figure 7—source data 4.Data associated with Figure 7I. Figure 7—source data 5.Data associated with Figure 7K.

We wondered if the VINE complex occupies endosomal membrane tubules as other SNX-BAR coat complexes do (Suzuki et al., 2021; van Weering et al., 2012; Zhang et al., 2021). To test this, we over-expressed both Vrl1 and GFP-Vin1 from the *ADH1* and *NOP1* promoters, respectively, and acquired images of GFP-Vin1 at 100ms intervals (Figure 7A). When compared to the endosomal marker Did2-mRuby2, we could observe GFP-Vin1 on tubular structures that eventually underwent scission and separated from the endosome (Figure 7A, B).

**Figure 7—figure supplement 1.**
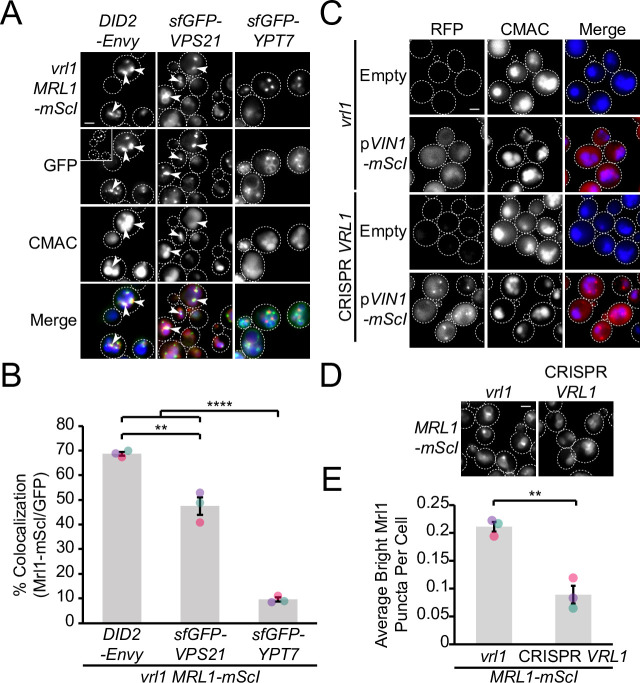
Vrl1 redistributes Mrl1 puncta from endosomes. (**A**) Mrl1-mScI puncta colocalize with Did2-Envy and sfGFP-Vps21-labeled endosomes, but not with sfGFP-Ypt7 puncta that label vacuolar sites. Insets are scaled to match other images in the same channel (see Materials and methods for details). (**B**) Quantification of colocalization as the percentage of Mrl1 puncta overlapping GFP puncta in *A*. One-way ANOVA with Tukey’s multiple comparison test; n=3, cells/strain/replicate ≥2286; **=p < 0.01, ****=p < 0.0001. (**C**) Localization of Vin1-mScI in BY4741 (*vrl1*) and an isogenic strain with the *vrl1* mutation corrected at the endogenous *VRL1* locus using CRISPR-Cas9 gene editing technology. Vin1-mScI localizes to puncta in a CRISPR-corrected *VRL1* strain. (**D**) Mrl1-mScI is redistributed from puncta in the CRISPR-corrected *VRL1* strain. (**E**) Quantification of large, bright Mrl1-mScI puncta per cell in *D*. Two tailed equal variance *t* test; n=3, cells/strain/replicate ≥1183; **=p < 0.01. Scale bars, 2 µm. Error bars report SEM. Figure 7—figure supplement 1—source data 1.Data associated with Figure 7—figure supplement 1B. Figure 7—figure supplement 1—source data 2.Data associated with Figure 7—figure supplement 1E.

The budding of VINE-coated endosomal tubules suggests the VINE complex could transport cargo proteins from this organelle. We examined the localization of several candidate cargo proteins in the presence and absence of Vrl1 (Figure 7C), including the mannose 6-phosphate receptor (MPR) homolog Mrl1, which had a strong DHFR interaction score with Vrl1 (Z=7.7; Figure 1F), and two proteins that require other SNX-BARs for their transport (Seaman et al., 1998; Seaman et al., 1997; Suzuki and Emr, 2018; Suzuki et al., 2021). We found that *VRL1* expression had no significant effect on the localization of the retromer cargo Vps10 or the Snx4 cargo Atg27 but caused a significant decrease in the number of bright Mrl1-GFP puncta per cell (61% decrease relative to *vrl1*, p<0.05; Figure 7D). The bright Mrl1 puncta in *vrl1* cells colocalize with the endosomal markers Did2-Envy and GFP-Vps21 (69% and 48%, respectively), but not with the vacuolar marker GFP-Ypt7 (9.6%, Figure 7—figure supplement 1A, B), suggesting that Vrl1 redistributes Mrl1 from endosomes. Vrl1 GEF activity was not required for this effect (p<0.05; Figure 7E, F), indicating that Vrl1 does not alter Mrl1 distribution by influencing the local activity of endosomal Rabs. Correction of the *vrl1* frameshift mutation using CRISPR-Cas9 gene editing technology restored the punctate localization of Vin1 (Figure 7—figure supplement 1C) and caused a similar change in Mrl1 localization (p<0.01; Figure 7—figure supplement 1D, E). This Vrl1-dependent change in Mrl1 distribution suggests that the VINE complex directly or indirectly regulates the Mrl1 intracellular trafficking itinerary.

The VINE complex could redistribute Mrl1 by recognizing sequences in its cytosolic tail or bind another protein that interacts with the MPR-like lumenal domain of Mrl1. A Vps10 mutant missing its cytoplasmic tail (Vps10^ΔCt^) lacks sorting signals and is transported to the vacuolar membrane and lumen (Bean et al., 2017; Cereghino et al., 1995; Cooper and Stevens, 1996). We hypothesized that if the Mrl1 tail contains a signal for VINE-mediated sorting, transplanting it onto Vps10^ΔCt^ (Vps10^ΔCt^-Mrl1^Ct^; Figure 7G) would confer VINE-dependent effects. We found that expression of *VRL1* did not alter the localization of WT Vps10 or Vps10^ΔCt^, the latter of which was targeted to the vacuolar membrane (Figure 7H). Vps10^ΔCt^-Mrl1^Ct^ localized to perivacuolar puncta and the vacuolar membrane in *vrl1* cells, indicating the presence of VINE-independent sorting signals in the Mrl1 tail (Figure 7H). *VRL1* expression caused a small but significant decrease in the proportion of cells displaying punctate Vps10^ΔCt^-Mrl1^Ct^ (14% decrease relative to *vrl1*, p<0.001; Figure 7I), suggesting that the Mrl1 cytoplasmic tail is sufficient to confer VINE-mediated redistribution.

In cells with functional VINE, Mrl1 is prominently localized to the vacuole membrane but accumulates at endosomes in its absence. This is reminiscent of the vacuolar membrane protein Atg27, which follows an AP-3 dependent Golgi-vacuole-endosome recycling loop that relies on the sequential action of two sorting nexins: Snx4, which transports Atg27 from vacuoles to the endosome, and retromer, which controls its endosome-to-Golgi transport (Eising et al., 2022; Segarra et al., 2015; Suzuki and Emr, 2018). In cells lacking retromer, Atg27 accumulates at endosomes due to continued recycling by Snx4. We observed Mrl1-mScI primarily at the vacuolar membrane in a *snx4*∆ strain (Figure 7J), which coincided with a significant decrease in endosomal Mrl1-mScI (p<0.001; Figure 7K), suggesting that like Atg27, Mrl1 is transported from the vacuole to the endosome by Snx4-containing coat complexes. Because VINE is present on budding endosomal tubules and depletes Mrl1 from endosomes, we hypothesize that VINE is required for a subsequent endosome-to-Golgi retrograde transport step. Taken together, these results support a model where VINE enhances the recycling of the cargo protein Mrl1 at endosomes (Figure 7L).

## Discussion

We have identified a novel endosomal SNX-BAR complex composed of the VPS9-domain GEF Vrl1 and the Vps5 paralog Vin1 which we have named the VINE complex. Our work suggests that VINE forms a novel endosomal coat with the potential to sort a unique set of cargo proteins that includes the mannose 6-phosphate receptor-like protein Mrl1.

### Divergent N-terminal sequences in paralogous SNX-BARs specify complex formation

The function of the Vps5-related SNX-BAR protein Vin1 was not previously known. We found that in the absence of Vrl1, Vin1 is unstable and displaced to the cytosol suggesting that it functions solely as a member of the VINE complex. Vin1 and Vrl1 are both predicted to have PX-BAR domains and dimerize through a canonical BAR-BAR interface, yet this interaction is not the primary driver of Vrl1-Vin1 association. Instead, we found that a short sequence within the unstructured N-terminal extension of Vin1 binds specifically to the AnkRD of Vrl1, and this is necessary for selective incorporation into the VINE complex. Our work suggests that VINE assembly requires two inputs: a strong interaction involving the Vin1 N-terminus and a weak interaction between the BAR domains that may occur primarily at the endosomal membrane.

The Vin1 paralog Vps5 also has an unstructured N-terminus that is critical for its assembly with the retromer complex and binds to a conserved patch on the Vps29 subunit (Collins et al., 2005; Seaman and Williams, 2002). Moreover, the interaction between Vps5 and Vps17 PX-BAR domains requires chemical crosslinkers to detect in detergent-solubilized lysates (Horazdovsky et al., 1997), suggesting the interaction is weak or mediated largely by hydrophobic contacts. This supports a model for retromer assembly that parallels that of the VINE complex, where the Vps5 N-terminus makes critical interactions with other retromer subunits and drives the assembly of the Vps5-Vps17 BAR-BAR dimer through an avidity effect.

Vin1 and Vps5 arose from a single ancestral gene during a whole-genome duplication (WGD) event (Byrne and Wolfe, 2005; Wolfe and Shields, 1997), and subsequently diverged to assume new roles in the VINE and retromer complexes, respectively. In pre-WGD species, the single ancestral form of Vps5 must partner with both Vrl1 and Vps17, which requires some promiscuity in BAR-BAR pairing. This is consistent with our structural modeling, which predicted a variety of pairings between the PX-BAR domains of Vps5, Vps17, Vrl1 and Vin1, while the PX-BAR protein Mvp1 was predicted to form only homodimers, consistent with in vivo observations (Suzuki et al., 2021).

van Weering et al., 2012 have proposed a lock and key model to explain the specificity of SNX-BAR pairing. In this model, paired charges within the hydrophobic BAR-BAR interface enforce the specificity of BAR-BAR interactions, and loss of these charged residues results in more promiscuous BAR pairing. Promiscuous BAR-BAR coupling could explain why interactions mediated by the N-termini of Vin1 and Vps5 are needed to specify complex assembly.

New functions have been uncovered for the extended N-termini of other SNX-BAR proteins, suggesting these extended regions have previously unappreciated regulatory roles (Shortill et al., 2022). SNX1, which is the human homolog of Vps5 and Vin1, recognizes SNX5 (or its homolog SNX6) through BAR-BAR interactions based on lock-and-key charge pairing, and engages with other complexes, including SNX27 (Simonetti et al., 2022; Yong et al., 2021) and the retromer subunit VPS29 (Swarbrick et al., 2011), through its unstructured N-terminal domain. Thus, the N-termini of the SNX1/Vps5/Vin1 family of SNX-BAR proteins have diversified to bind different proteins and participate in different sorting complexes.

### The VINE complex is both a SNX-BAR coat and a VPS9-domain GEF

The VINE complex is the first described SNX-BAR coat to possess a VPS9 domain-containing subunit. Retromer binds the VPS9-domain GEFs Vps9 and Muk1, which redundantly activate Rab5-like GTPases to stimulate PI3P production at endosomes (Bean et al., 2015). One benefit of wiring SNX-BARs to VPS9-domain GEFs could be to generate a local enrichment of PI3P that enhances SNX-BAR assembly. Indeed, we find that VINE localization requires its own GEF activity in a strain lacking other VPS9-domain GEFs.

The human Vrl1 homolog VARP also contains a VPS9 domain and associates with retromer (Hesketh et al., 2014), albeit through a distinct interaction involving a motif that is not present in Vrl1 (Crawley-Snowdon et al., 2020). This example of convergent evolution suggests that the linking of retromer to the activation of endosomal Rabs has an important and conserved role. VARP activates Rab21, which is related to Rab5 (Stenmark and Olkkonen, 2001) and interacts with PI3K in a proximity-based assay (Del Olmo et al., 2019), although it has not yet been shown to stimulate PI3K activity. Because endosomal Rab GTPases also recruit a variety of effectors including the conserved tethering complexes Rabenosyn-5/Vac1 and CORVET (Cabrera et al., 2013; Christoforidis et al., 1999; Peplowska et al., 2007; Peterson et al., 1999) further work is required to clarify the conserved functional link between VPS9-domain GEFs and SNX-BAR sorting complexes.

### VINE forms endosomal transport carriers and regulates cargo distribution

Our work suggests that VINE may also be a novel sorting complex that acts in a pathway-specific manner, thus joining the group of SNX-BAR complexes that promote independent sorting pathways from the endosome or vacuole in yeast (Ma and Burd, 2020; Suzuki et al., 2021). As VINE can be visualized at budding endosomal structures, we hypothesize that it recycles cargo from this organelle. We identified the MPR-related protein Mrl1 as a candidate VINE cargo. Although the function of Mrl1 is unclear, there is evidence that it works jointly with Vps10 to enhance the transport or maturation of some vacuolar proteases (Whyte and Munro, 2001). Restoring VINE function alters the steady state localization of Mrl1 and could modulate the rate at which it delivers proteins to the vacuole. Because Mrl1 contributes to protease delivery in a *vrl1* mutant strain (Whyte and Munro, 2001), it is likely that other SNX-BAR complexes redundantly regulate Mrl1 sorting. Indeed, recent studies from our group and others have identified redundant cargo sorting roles of yeast SNX-BARs (Bean et al., 2017; Best et al., 2020; Suzuki et al., 2021).

Further work will be needed to determine if VINE binds directly to the Mrl1 cytosolic domain to direct its sorting as VARP does with VAMP7 (Crawley-Snowdon et al., 2020; Hesketh et al., 2014; Schäfer et al., 2012). VARP has additional domains not found in Vrl1 and it is not yet clear if the sorting functions of VARP are conserved in Vrl1. It is instead possible that VINE promotes the redistribution of Mrl1 indirectly, by regulating other processes such as those controlled by TORC1. Recent studies have placed functional pools of the TORC1 machinery at endosomes (Hatakeyama et al., 2019) and the TORC1-activating EGO/Ragulator complex is proximal to VINE based on our DHFR screening results. Vin1 was also recently reported to regulate TORC1 in strains carrying the *vrl1* mutation (Goyal et al., 2022), suggesting a connection between VINE and TORC1. While a cargo-centric interpretation of our results is consistent with known functions of SNX-BAR complexes, a more detailed investigation is required to clarify the role of VINE and to understand metabolic implications of restoring VINE function.

Importantly, almost all studies on endosomal trafficking and signaling have been performed in strains that have a *vrl1* mutation and lack the VINE complex, and we anticipate that other VINE-regulated cargoes may exist. Some proteins that are recycled by SNX-BAR-dependent pathways, such as phospholipid flippases, are themselves important for organelle function or signaling (Dalton et al., 2017; Liu et al., 2008). In the absence of functional VINE, such proteins may be missorted and/or have altered properties. Restoring VINE may therefore restore cellular processes and reveal new biology.

## Materials and methods

**Key resources table:** 

Reagent type (species) or resource	Designation	Source or reference	Identifiers	Additional information
Antibody	Anti-HA (Mouse monoclonal)	Sigma-Aldrich	H9658; HA-7	WB (1:1000)
Antibody	Anti-HA (Mouse monoclonal)	Covance	MMS-101R; HA.11	WB (1:1000)
Antibody	Anti-GFP (Mouse monoclonal)	Roche	11-814-460-001	WB (1:1000)
Antibody	Anti-Pgk1 (Mouse monoclonal)	Invitrogen	AB_2532235; 22C5D8	WB (1:1000)
Antibody	Anti-GFP (Rabbit polyclonal)	Eusera	EU2	CoIP
Antibody	Anti-HA (Rabbit polyclonal)	AbCam	Ab9110	CoIP
Antibody	HRP-Anti-Mouse (Goat polyclonal)	Jackson	115-035-146	WB (1:20,000)
Other	nProtein A Sepharose 4 Fast Flow	Cytiva	17528004	CoIP
Other	Amersham Hyperfilm	GE Healthcare	28906839	Chemiluminescent film
Other	Amersham ECL	Cytiva	GERPN2209	Chemiluminescent reagent
Other	Amersham ECL Prime	Cytiva	GERPN2232	Chemiluminescent reagent
Other	Yeast/Fungal ProteaseArrest	GBiosciences	786–435	Yeast protease inhibitor; 100 X
Other	Concanavalin A	Sigma-Aldrich	C2010	Yeast live-cell imaging preparation
Other	FM4-64	Invitrogen	T3166	Vacuolar Rim Stain; 4 μM
Other	CMAC	Setareh Biotech	6627	Vacuole Stain; 100 µM
Chemical compound, drug	Methotrexate	Enzo Life Sciences	ALX-440–045 G001	DHFR inhibitor; 200 μg/mL, DMSO
Software, algorithm	CellProfiler	Lamprecht et al., 2007		Yeast colony array image analysis
Software, algorithm	MetaMorph	MDS Analytical Technologies	Version 7.8	Automated image analysis
Software, algorithm	GraphPad Prism	GraphPad Software	Version 9.1.0	Statistical analysis
Software, algorithm	ImageJ	NIH		Band densitometry
Software, algorithm	ColabFold AlphaFold2 Advanced	Jumper et al., 2021; Mirdita et al., 2022		Protein structure and binding prediction software

### Yeast strains and plasmids

Yeast strains and plasmids used in this study are described in Supplementary files 4 and 5, respectively. Yeast strains were built in the BY4741 strain background using homologous recombination-based integration unless otherwise indicated. Gene deletions, promoter exchanges and tags were confirmed by colony PCR and either western blot or fluorescence microscopy where possible. Plasmids were built by homologous recombination in yeast, recovered in *Escherichia coli* and confirmed by sequencing.

### Bioinformatic analysis of protein folding and sequence conservation

Prediction of protein structure and binding interfaces was performed using Phyre2 (Kelley et al., 2015) and the ColabFold AlphaFold2 advanced server (Jumper et al., 2021; Mirdita et al., 2022) with default settings (https://colab.research.google.com/github/sokrypton/ColabFold/blob/main/beta/AlphaFold2_advanced.ipynb#scrollTo=bQe3KeyTcv0n). ColabFold complex prediction confidence is reported as a pTMscore, which is a predicted template modeling score (TM-score; Zhang and Skolnick, 2004) derived from the predicted alignment error (PAE; Jumper et al., 2021). Vrl1 amino acid sequences were from the *S. cerevisiae* strain RM11-1a. Orthologous sequences were obtained from the OrthoDB database (Kriventseva et al., 2019), aligned using the EMBL-EBI Multiple Sequence Comparison by Log-Expectation (MUSCLE) tool (https://www.ebi.ac.uk/Tools/msa/muscle) and presented using Jalview (http://www.jalview.org). Protein sequence conservation was mapped to predicted structure using ConSurf (https://consurf.tau.ac.il; Ashkenazy et al., 2016).

### DHFR protein fragment complementation assay and ontology analysis

A MATa strain containing a plasmid that expresses the Vrl1-DHFR[1,2] (DHFR^Nt^) fusion from the *ADH1* promoter, or the p*ADHpr-VRL1(1–465)-DHFR^Nt^* control, was crossed into a library of MATα strains (n = ~4300) expressing proteins fused to DHFR[3] (DHFR^Ct^; Tarassov et al., 2008). Diploids were subjected to two rounds of double mutant selection followed by two rounds of selection on media containing 200 µg/ml methotrexate, in 1536 arrays. Manipulations were carried out using a BM3-BC pinning robot (S&P Robotics inc, Toronto, Canada). Colony area was analyzed using CellProfiler (Lamprecht et al., 2007) after 8 days at 30 °C. Z-scores were generated using median colony area from two technical replicates for each Vrl1-prey combination. Functional analysis of Vrl1 DHFR interactors (Z>2) was performed using the Gene Ontology (Ashburner et al., 2000; Gene Ontology Consortium, 2021) GO Enrichment Analysis tool (Mi et al., 2019).

### Fluorescence microscopy and automated image analysis

Yeast cells were diluted from overnight cultures in fresh synthetic dextrose-based media (SD) and incubated at 30 °C for ~4 hr or until they reached an optical density of ~0.4–0.7 OD_600_ unless otherwise indicated. Log phase yeast were transferred to concanavalin A-treated 96-well glass bottom plates (Eppendorf, Hamburg, Germany) and imaged using a DMi8 microscope (Leica Microsystems, Wetzlar, Germany) equipped with an ORCA-flash 4.0 digital camera (Hamamatsu Photonics, Shizuoka, Japan) and a high-contrast Plan-Apochromat 63 x/1.30 Glyc CORR CS immersion lens (Leica Microsystems, Wetzlar, Germany). Image acquisition and processing was performed using the MetaMorph 7.8 software package (MDS Analytical Technologies, Sunnyvale, California). Yeast vacuoles were labelled with 100 µM CMAC (Setareh Biotech, San Jose, California) or 4 µM FM4-64 (Invitrogen, Waltham, Massachusetts) for 30 minutes at 30 °C. Dye-treated cells were washed once in SD media prior to imaging.

Linear intensity scale changes were uniformly applied to all images of a given fluorophore in an experimental set using MetaMorph 7.8 (MDS Analytical Technologies, Sunnyvale, California). For very dim or bright signals that could not be identically scaled, uniformly applied brightness settings are shown as insets and custom settings were used to show protein localization in the full-size image. Images were prepared for presentation using Photoshop CC 2020 (Adobe, San Jose, California) and Illustrator CC 2020 (Adobe, San Jose, California). Quantification was performed on unscaled raw images with scripted MetaMorph 7.8 journals (MDS Analytical Technologies, Sunnyvale, California). The Count Nuclei feature was used to filter out dead cells and identify live cells based on intensity above local background (IALB). The Granularity feature was used to identify puncta in a dead cell-masked intermediate image based on IALB. Masking functions were performed using the Arithmetic function with Logical AND.

### Coimmunoprecipitation, western blotting, and spheroplasting

For western blot-based stability assays, yeast cells were grown to log phase in SD media at 30 °C and 10 OD_600_/mL equivalents of cells were harvested and stored at –80 °C. Cells were thawed and lysed by vortexing in 100 µL of Thorner buffer (8 M Urea, 5% SDS, 40 mM Tris-Cl (pH 6.4), 1% beta-mercaptoethanol and 0.4 mg/mL bromophenol blue) with ~100 µL of acid-washed glass beads/sample at 70 °C for 5 minutes. Lysates were centrifuged at 14,000 RPM for 30 s and separated on 8% SDS-PAGE gels followed by western blotting with mouse anti-HA (H9658, Clone HA-7, Sigma-Aldrich) or anti-PGK1 monoclonal antibodies (AB_2532235, 22C5D8, Invitrogen), and secondary polyclonal goat anti-mouse antibodies conjugated to horseradish peroxidase (115–035-146; Jackson ImmunoResearch Laboratories).

For CoIPs, yeast cells were grown to log phase in SD media at 30 °C and 75 OD_600_/mL equivalents of cells were incubated in 50 mM Tris-Cl with 10 mM DTT (pH 9.5) for 15 minutes at room temperature and digested in spheroplasting buffer (1.2 M sorbitol, 50 mM KH_2_PO_4_, 1 mM MgCl_2_ and 250 µg/ml zymolase at pH 7.4) at 30 °C for 1 hr. Spheroplasts were washed twice with 1.2 M sorbitol, frozen at –80 °C, then incubated in 500 µL of lysis buffer (0.1% Tween-20, 50 mM HEPES, 1 mM EDTA, 50 mM NaCl, 1 mM PMSF and 1 x fungal ProteaseArrest, pH 7.4) at room temperature for 10 minutes. A total of 50 µL volumes of lysate were collected for each sample and mixed with 2 x Laemmli buffer (4% SDS, 20% glycerol, 120 mM Tris-Cl (pH 6.8), 0.01 g bromophenol blue and 10% beta-mercaptoethanol) for western analysis while remaining lysates were incubated with either a polyclonal rabbit anti-GFP (EU2, Eusera) or a polyclonal rabbit anti-HA antibody (ab9110, Abcam) at 4 °C for 1 hr. Antibody-treated samples were next incubated with Protein A Sepharose beads (Cytiva) at 4 °C for 1 hr. Beads were washed 3 x in lysis buffer before being resuspended in 50 µL of Thorner buffer and heated at 80 °C for 5 minutes. Western blotting of proteins separated on 8% SDS-PAGE gels was carried out with monoclonal mouse anti-HA (H9658, Clone HA-7, Sigma-Aldrich), monoclonal mouse anti-HA (MMS-101R; Covance) or monoclonal mouse anti-GFP antibodies (11–814–460-001; Roche) prior to secondary antibody treatment with polyclonal goat anti-mouse conjugated to horseradish peroxidase (115–035-146; Jackson ImmunoResearch Laboratories). Blots were developed with Amersham ECL (GERPN2209, Cytiva) or Amersham ECL Prime (GERPN2232, Cytiva) chemiluminescent western blot detection reagents and exposed using Amersham Hyperfilm ECL (GE Healthcare). Densitometry of scanned films was performed using ImageJ (Schneider et al., 2012).

### Correction of genomic *vrl1* mutation using CRISPR-Cas9

A plasmid containing the Cas9 enzyme and a single guide RNA (sgRNA) targeting the *VRL1*-disrupting *yml003w* mutation was pre-cloned using small fragment golden gate assembly (Marillonnet and Grützner, 2020). BY4741 was co-transformed with linearized split *URA3* marker Cas9-sgRNA(*VRL1*) plasmid and PCR product containing the corrected *VRL1* sequence. Ura^+^ colonies were sequenced and an isolate with intact *VRL1* was used for experiments.

### Statistical analysis of quantitative data

Statistical tests were performed using GraphPad Prism 9.1.0 (GraphPad Software, San. Diego, California) as indicated in figure legends with the appropriate post-hoc tests. Normality of data was assumed but not formally tested and hypotheses were measured against a threshold of 95% confidence (or p<0.05). Graphs were made in Microsoft Excel 2019 (Microsoft, Redmond, Washington). Column charts represent the average value from biological replicates while scatter points represent data from individual replicates and are colored by replicate. Error bars report the standard error of the mean value.

## Data Availability

All data generated or analyzed during this study are included in the manuscript and supporting files; Supplementary File 1 contains data from a genome-wide protein proximity screen and Source Data files have been provided for all graphs and blots.

## Additional files

Supplementary file 1. Vrl1 DHFR Interactors. List of Z-scores from the Vrl1 DHFR screen.

Supplementary file 2. Vrl1 DHFR Ontology Enrichment. List of enriched ontology terms for Vrl1 DHFR interactors (Z>2).

Supplementary file 3. Yeast SNX-BAR Dimer Predictions. Results of pairwise yeast SNX-BAR prediction matrix generated using ColabFold.

Supplementary file 4. List of *Saccharomyces cerevisiae* strains used in this study.

Supplementary file 5. List of plasmids used in this study.

Transparent reporting form
