# Supplementary figures and images for: The VINE complex is an endosomal VPS9-domain GEF and SNX-BAR coat
Source: eLife. 2022 Aug 8;11:e77035. doi: 10.7554/eLife.77035 (PMC9507130; doi:10.7554/eLife.77035)

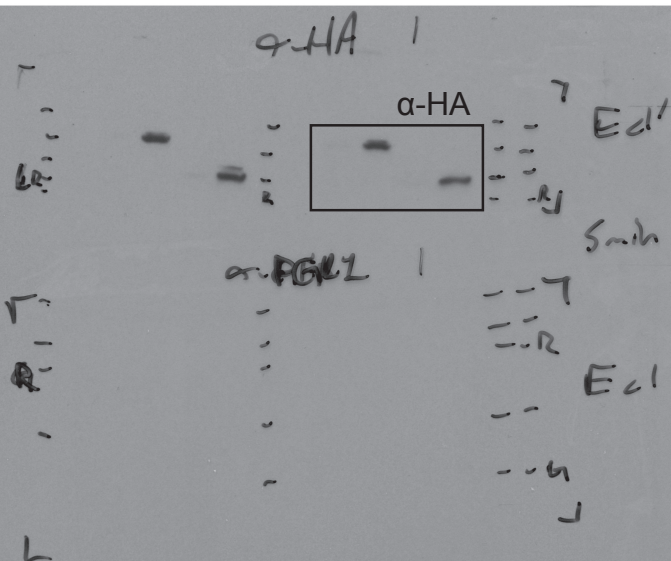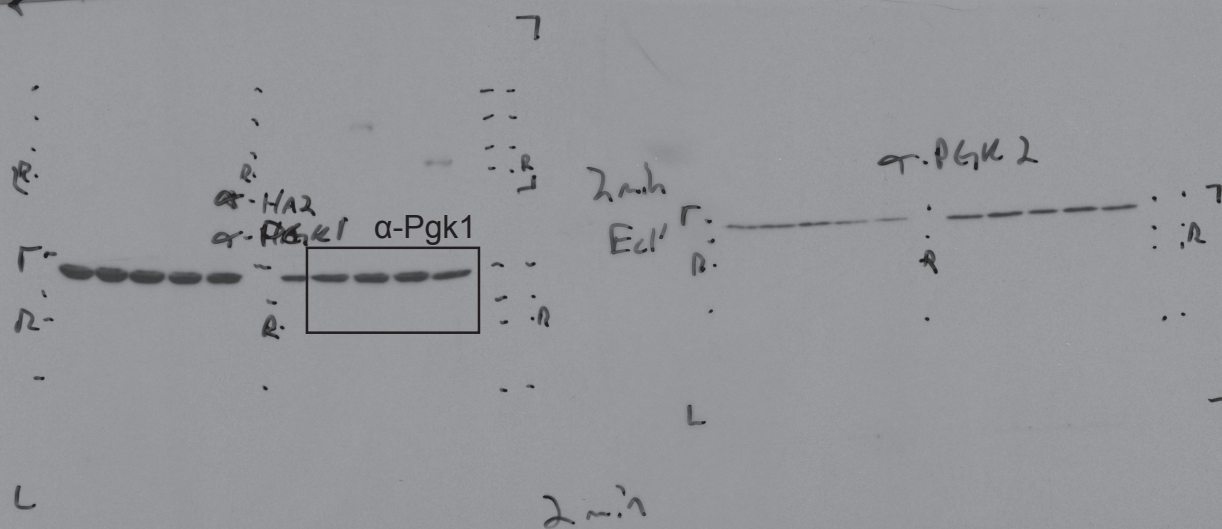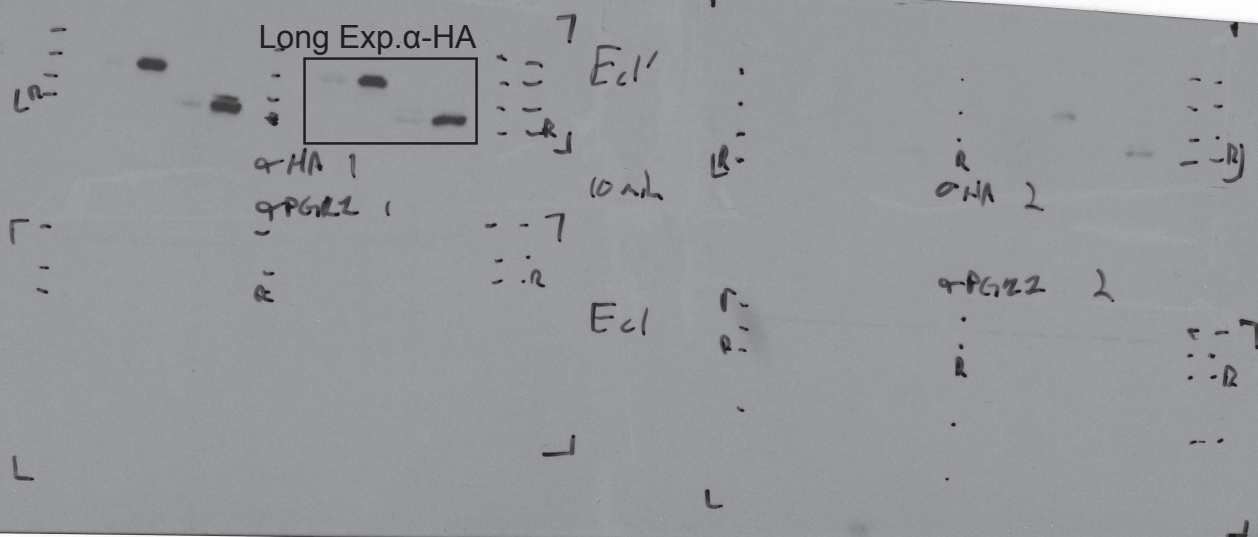

Supplement: Figure 2—source data 6. [file elife-77035-fig2-data6.zip › Figure 2D Uncropped Blots.pdf]

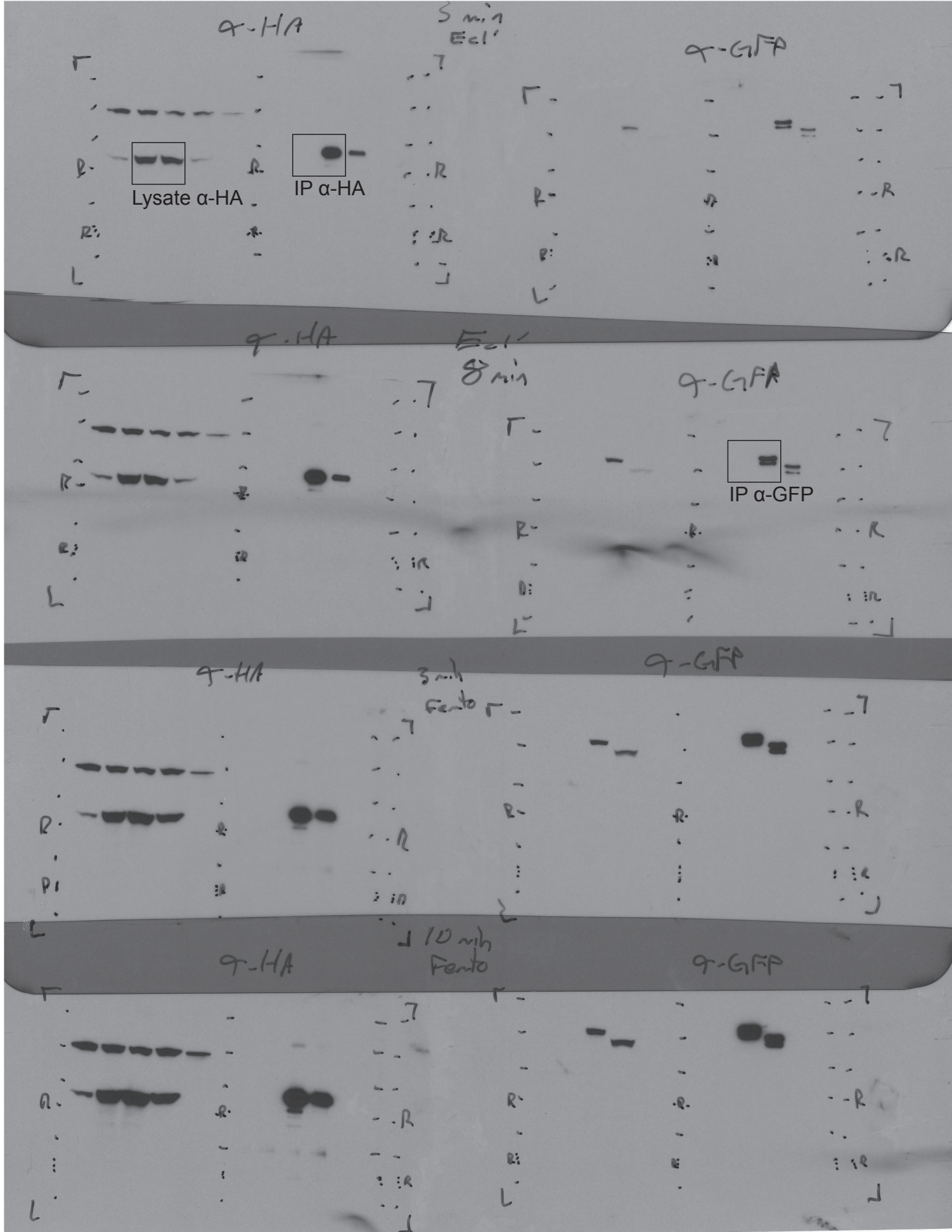

Supplement: Figure 2—source data 7. [file elife-77035-fig2-data7.zip › Figure 2F Uncropped Blots.pdf]

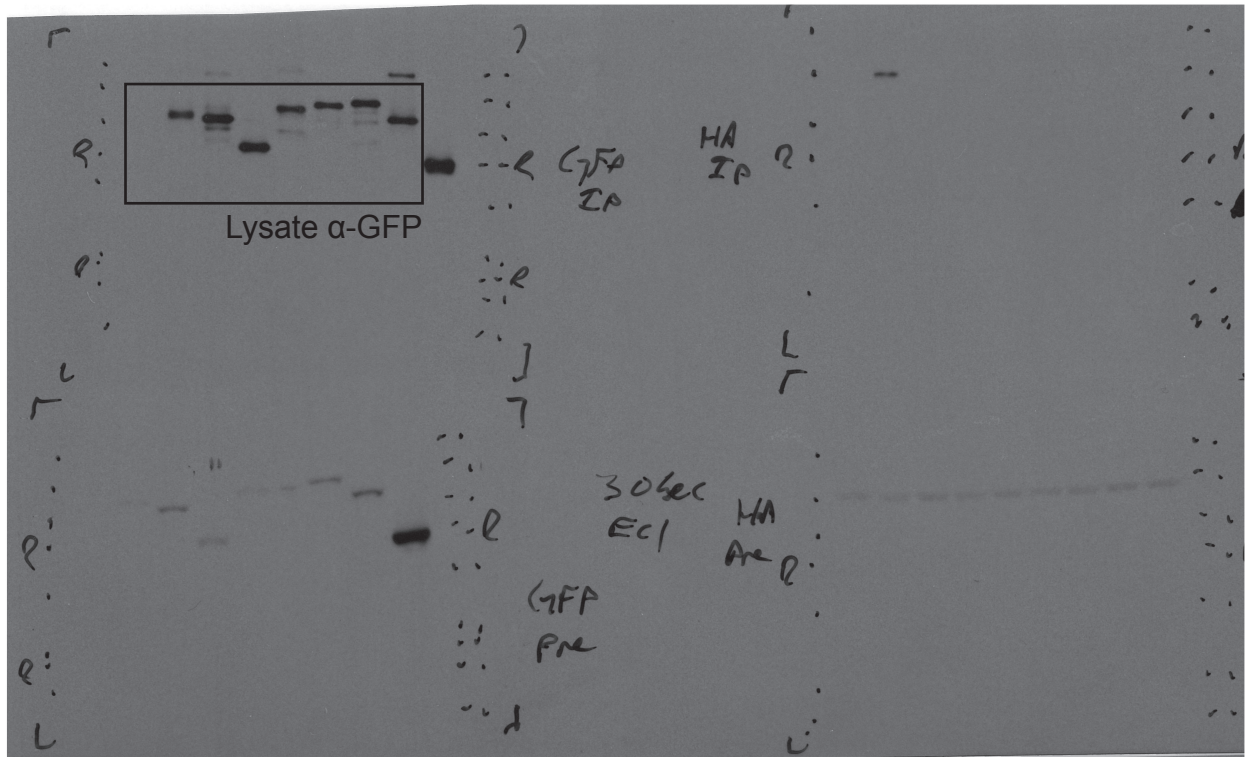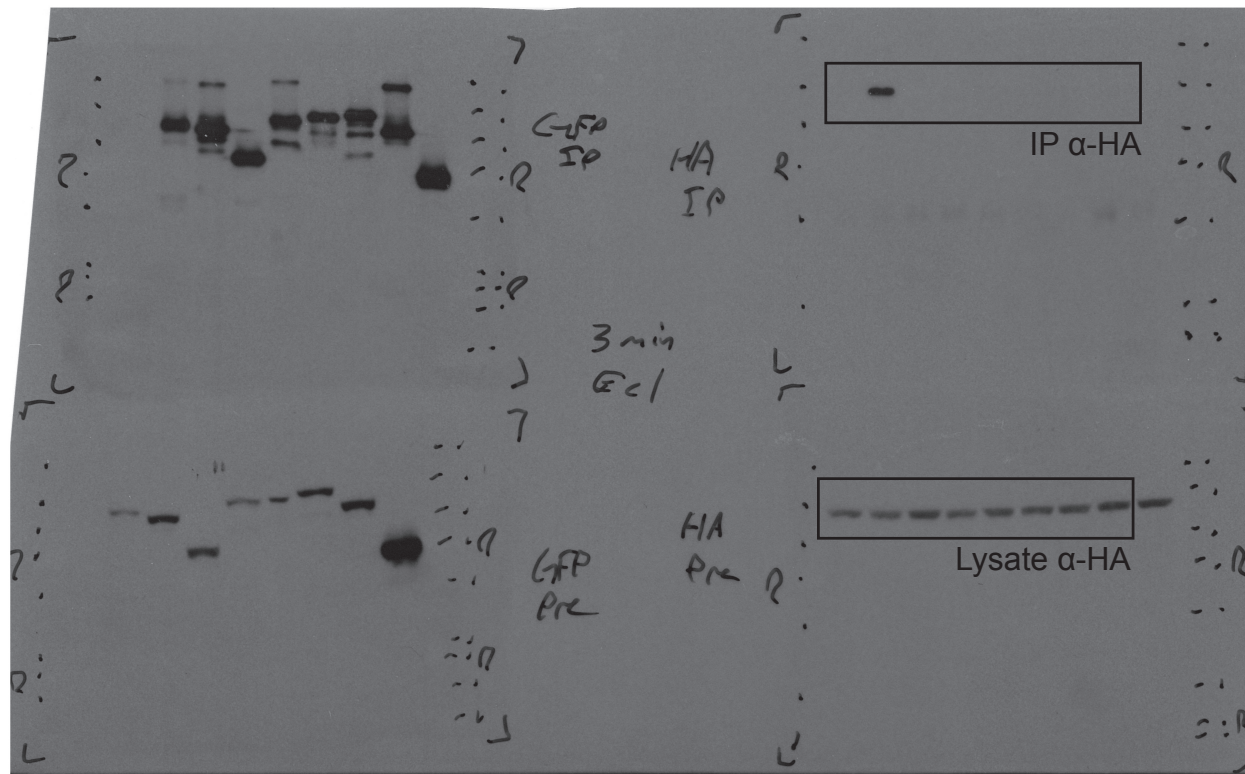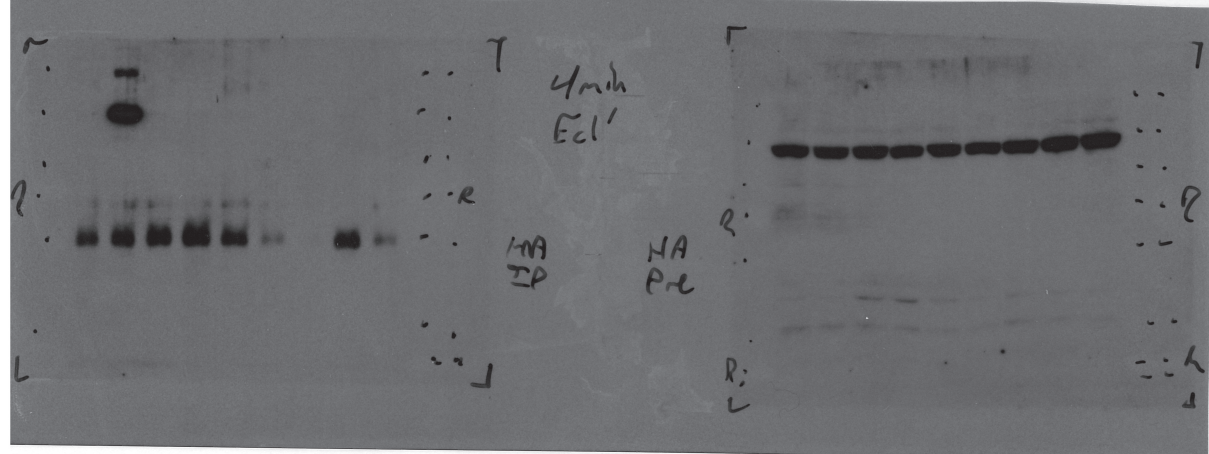

Supplement: Figure 3—source data 3. [file elife-77035-fig3-data3.zip › Figure 3C Uncropped Blots.pdf]

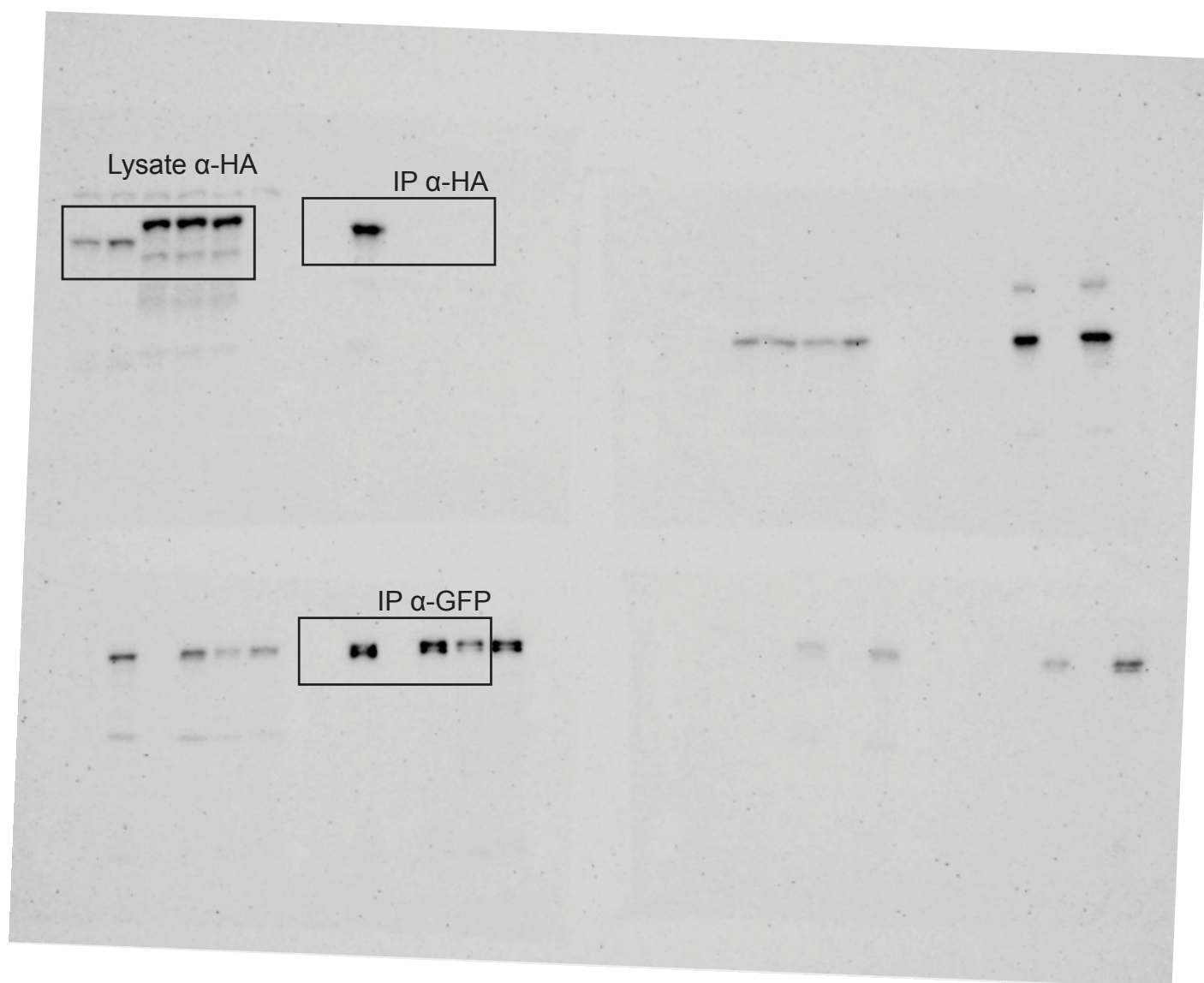

Supplement: Figure 3—source data 4. [file elife-77035-fig3-data4.zip › Figure 3D Uncropped Blots.pdf]

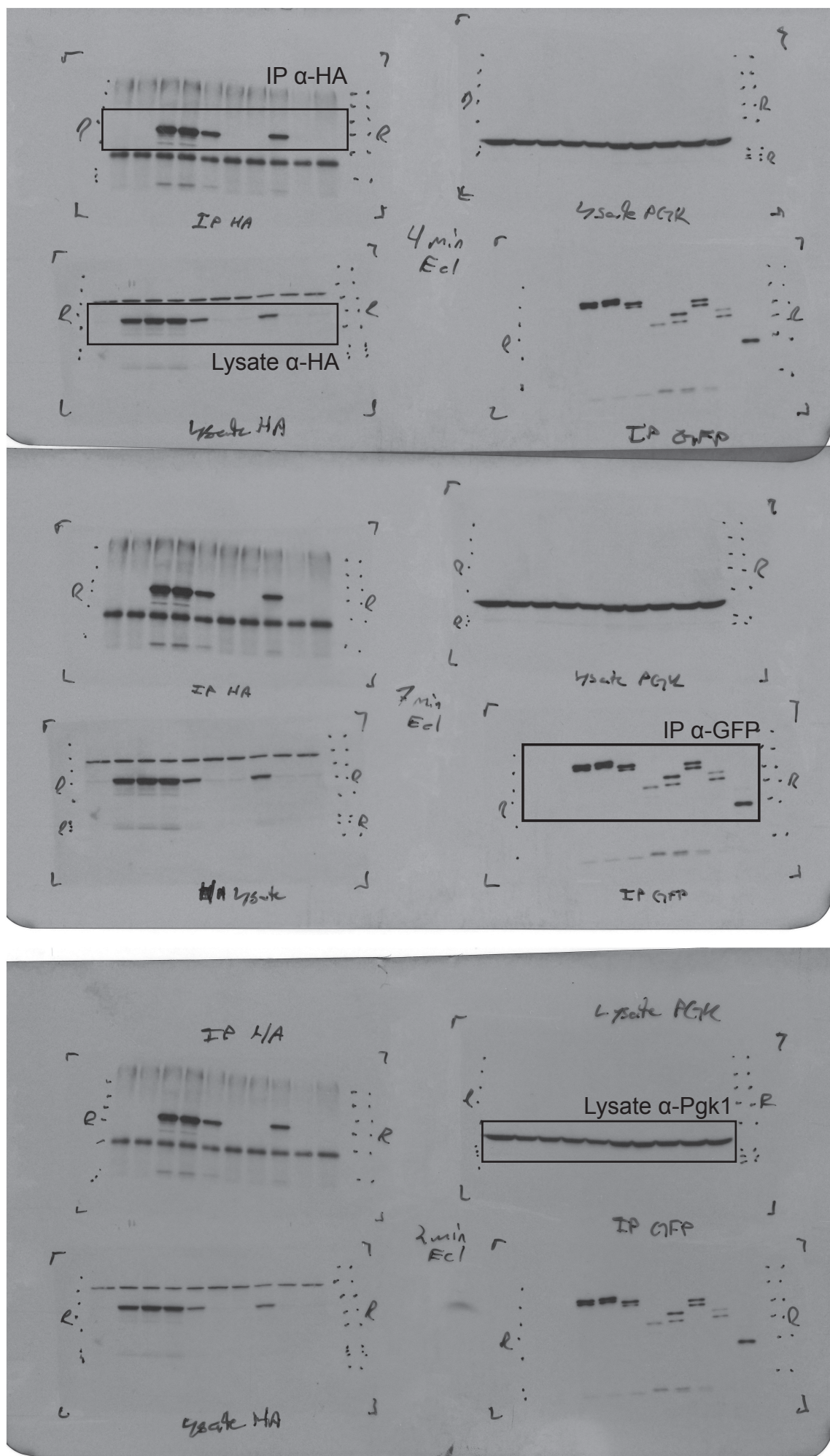

Supplement: Figure 3—source data 5. [file elife-77035-fig3-data5.zip › Figure 3I Uncropped Blots.pdf]

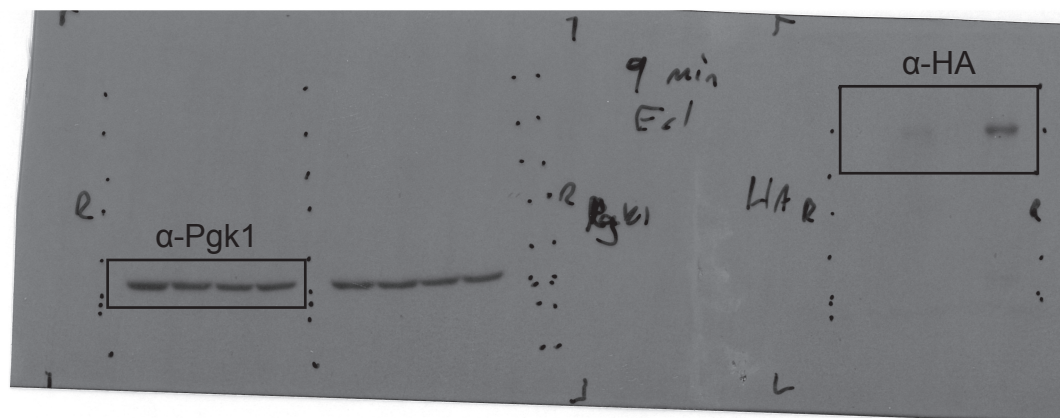

Supplement: Figure 3—figure supplement 2—source data 1. [file elife-77035-fig3-figsupp2-data1.zip › Figure 3 S2 Uncropped Blots.pdf]

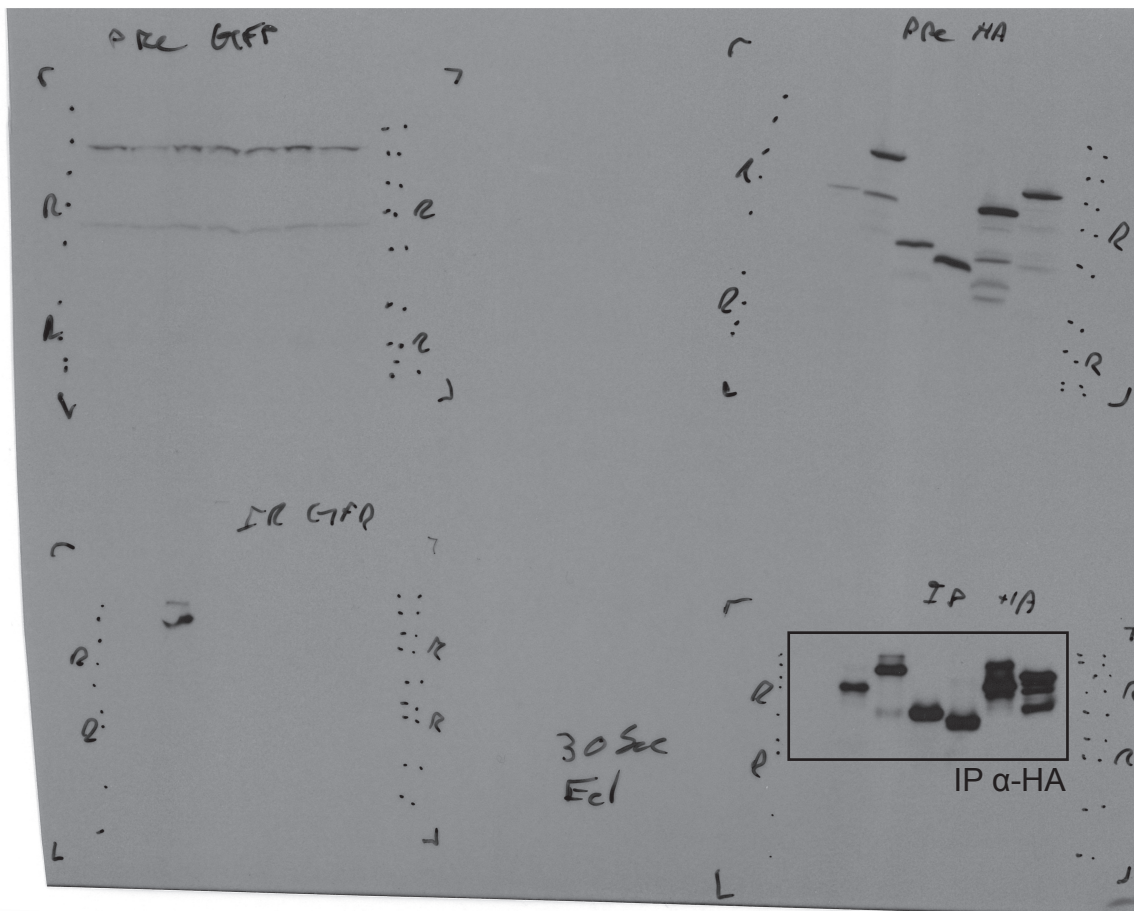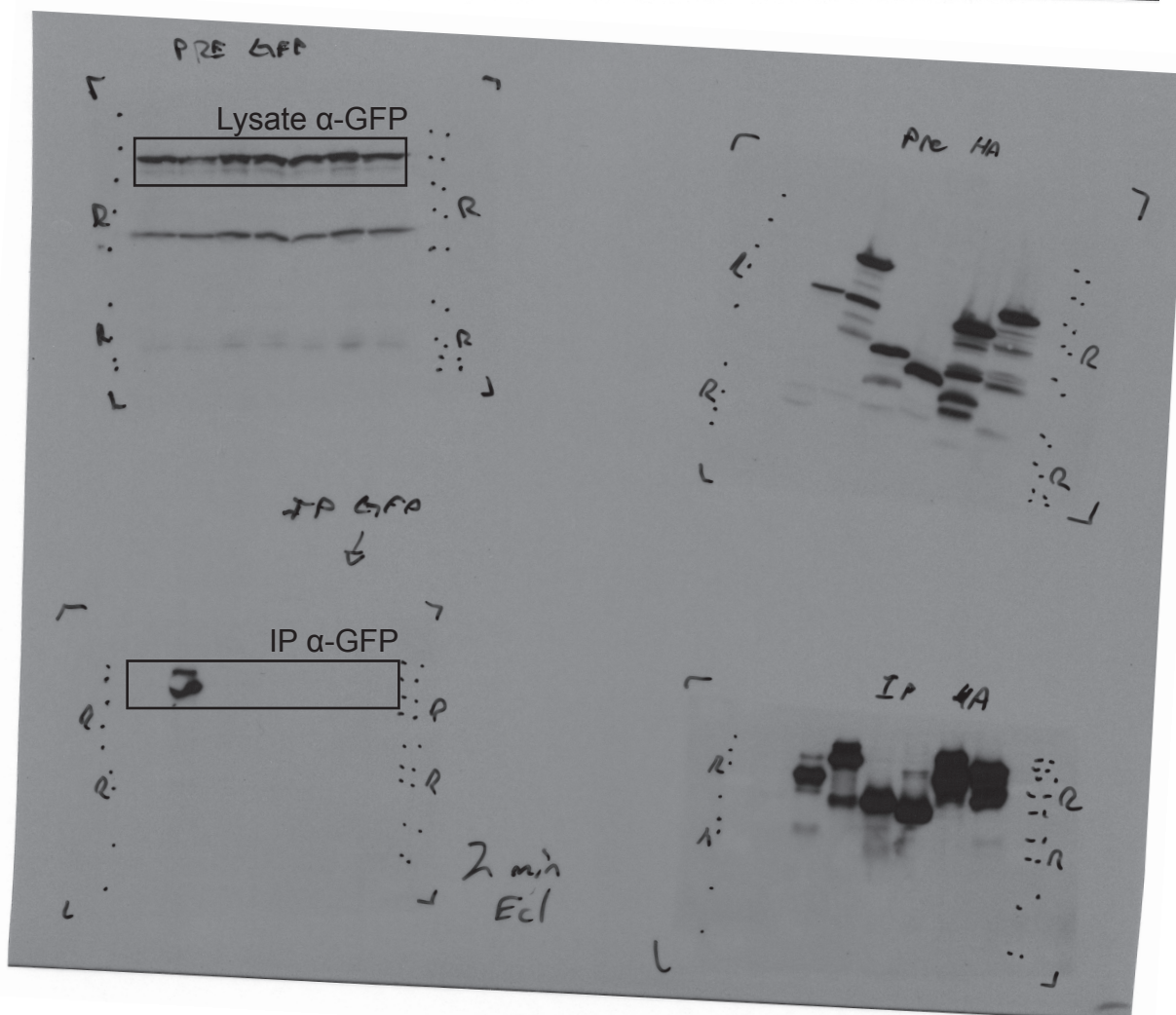

Supplement: Figure 3—figure supplement 3—source data 2. [file elife-77035-fig3-figsupp3-data2.zip › Figure 3 S3A Uncropped Blots.pdf]

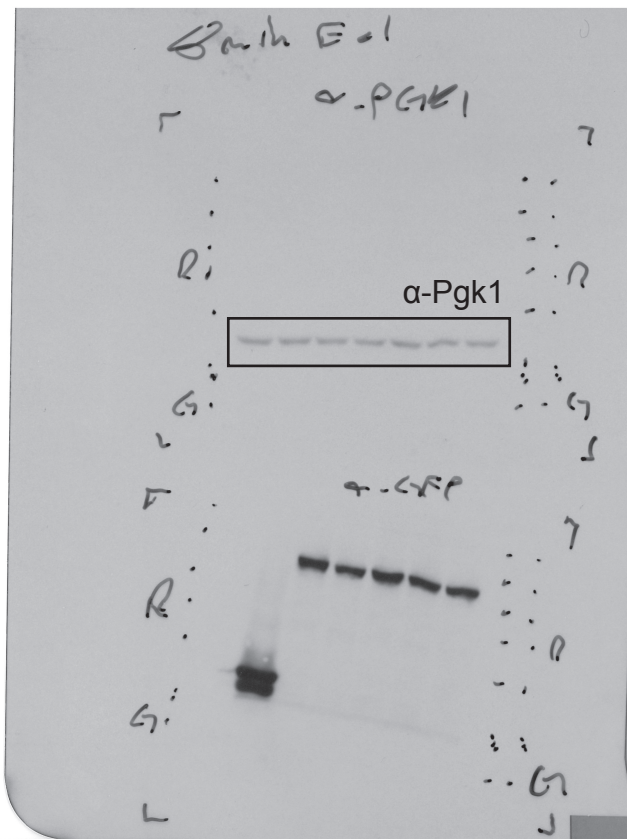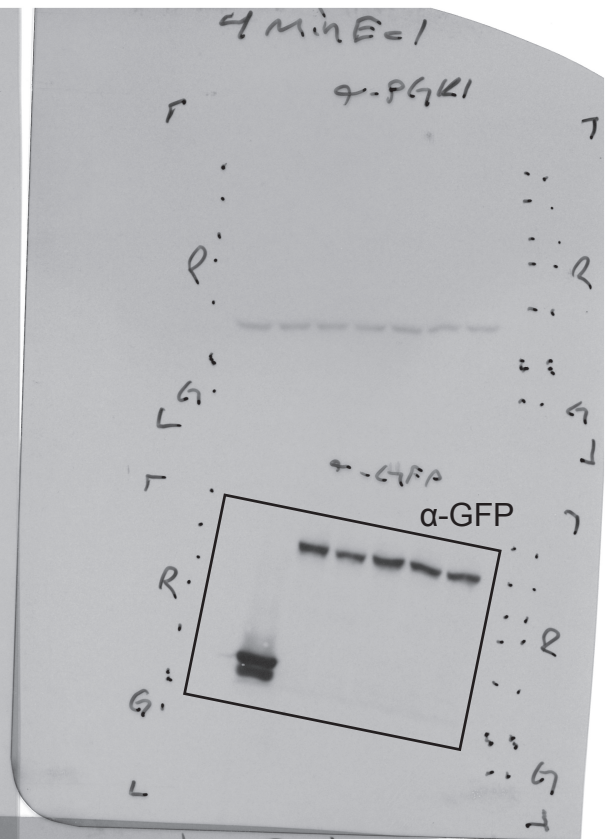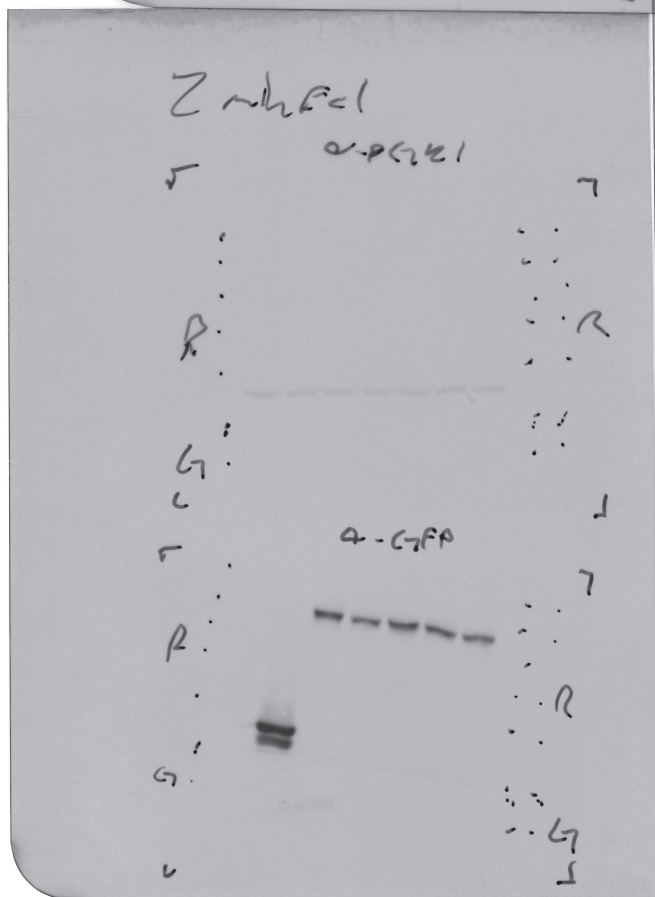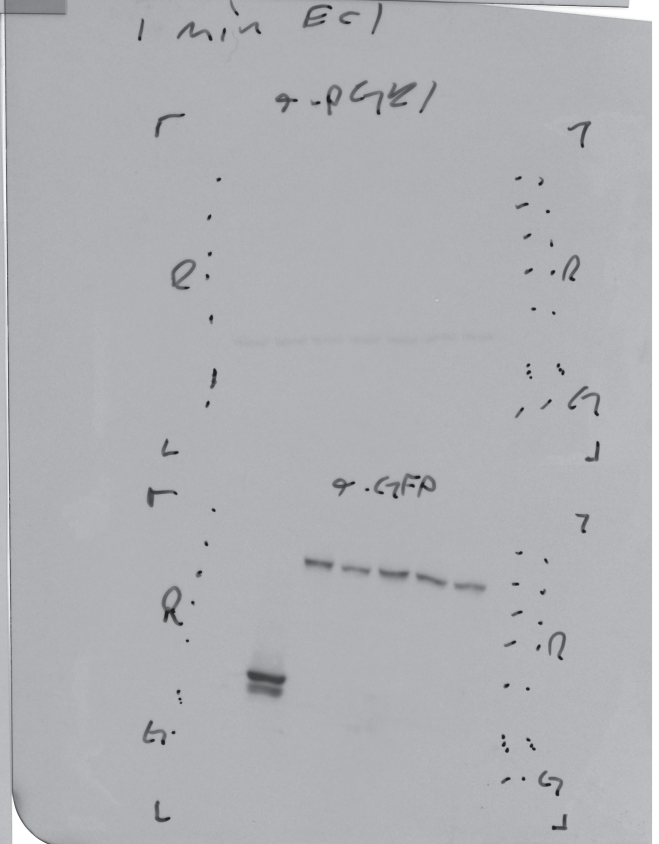

Supplement: Figure 5—figure supplement 2—source data 1. [file elife-77035-fig5-figsupp2-data1.zip › Figure 5 S2 Uncropped Blots.pdf]
