# Supplementary material for: The VINE complex is an endosomal VPS9-domain GEF and SNX-BAR coat
Source: eLife. 2022 Aug 8;11:e77035. doi: 10.7554/eLife.77035 (PMC9507130; doi:10.7554/eLife.77035)
Supplement: Peer review history

# Editor report: Editor's evaluation

**DOI:** 10.7554/eLife.77035.sa0

**Authors:** Suzanne R Pfeffer

**Affiliations:** https://ror.org/00f54p054 Stanford University School of Medicine United States

The SNX-BAR family of sorting nexins are a diverse group of dimeric proteins that form tubules from endosomal membranes where they also select protein cargoes for incorporation into these transport carriers. Here the authors describe a new SNX-BAR complex in budding yeast, named the VINE complex, that uniquely harbors a guanine nucleotide exchange factor (GEF) domain for Rab5-related small GTP-binding proteins. The authors' highly compelling data implicate VINE in endosomal membrane remodeling events and the sorting of a vacuolar hydrolase receptor and will be of broad interest to scientists studying membrane trafficking and receptor recycling.

# Decision letter

**DOI:** 10.7554/eLife.77035.sa1

**Affiliations:** https://ror.org/00f54p054 Stanford University School of Medicine United States; https://ror.org/02vm5rt34 Vanderbilt University United States; https://ror.org/00rqy9422 University of Queensland Australia

Our editorial process produces two outputs: i) public reviews designed to be posted alongside the preprint for the benefit of readers; ii) feedback on the manuscript for the authors, including requests for revisions, shown below. We also include an acceptance summary that explains what the editors found interesting or important about the work.

**Decision letter after peer review:**

Thank you for submitting your article "The VINE complex is a VPS9-domain GEF-containing SNX-BAR coat involved in endosomal sorting" for consideration by *eLife*. Your article has been reviewed by 2 peer reviewers, and the evaluation has been overseen by a Reviewing Editor and Suzanne Pfeffer as the Senior Editor. The following individuals involved in review of your submission have agreed to reveal their identity: Todd R Graham (Reviewer #1); Brett M Collins (Reviewer #2).

The reviewers have discussed their reviews with one another, and the Reviewing Editor has drafted this to help you prepare a revised submission.

Essential revisions:

1) Resolve questions related to Mrl1 localization with experimental data and text clarification (see below)

2) Model the complex between Vrl1's AnkRD and the peptide region from the Vin1 N-terminus, and test the model with a few mutants (see below).

*Reviewer #1 (Recommendations for the authors):*

My primary concern is with the Mrl1 localization data shown in Figure 6 and so most of my comments will focus on this issue.

1. The images presented for the vrl1 and VRL1 cells are very similar although the "GFP Puncta Per Cell" quantitation shows a strong reduction of puncta in cells with wild-type VRL1. A reduction in "bright" puncta is a subjective measure and could also be caused by lower expression levels of the MRL1-GFP or lateral changes in distribution around the vacuole membrane – not necessarily mislocalization from one organelle to another. The model in 6L argues for a role of VINE in sorting Mrl1 from endosomes to the Golgi, consistent with the function of other sorting nexins. If this is correct, I would expect cells with WT VRL1 would display a greater number of Mrl1-GFP puncta and the vrl1 mutant would display most of Mrl1 in the vacuole. The data shown in Figure 6C and D appears to indicate the opposite result. If the puncta are endosomes, the data would argue a role of VINE in the delivery of Mrl1 from endosomes to the vacuole. This is the conclusion stated in the abstract of the manuscript but the conclusion is reversed in the Figure 6L model. The authors should try to better define these Mrl1 puncta using less biased, more quantitative approaches to address potential changes in organelle localization. For example, do the Mrl1-GFP puncta co-localize with Did2-mRuby? Can the authors report a Manders coefficient between Did2-Ruby/Mrl1-GFP and Mrl1-GFP/Vph1-mCherry (vacuolar marker) in cells +/- Vrl1 that would support a VINE-dependent change in Mrl1 localization?

2. Mrl1-GFP appears to be concentrated at sites of membrane contact between vacuoles (vertices) and possibly between vacuoles and the prevacuolar endosome compartment. Could the vacuole/prevacuolar contact site be the observed Mrl1 puncta? Co-localization with Ypt7 could test this possibility. Figure 1 reports an interaction between Vrl1 and Vps11/Vps18, part of the core HOPS complex which is found at these pre-fusion contact sites. It seems possible that VINE binding to HOPS and/or Mrl1 somehow prevents the concentration of Mrl1 at these contact sites and changes the lateral distribution of Mrl1 in the vacuolar membrane.

3. Transplantation of the Mrl1 C-terminal tail onto Vps10∆CT caused a change in the localization pattern from uniform vacuole membrane with some concentration at vertices, to a localization pattern that looks more like Mrl1-GFP in the vrl1 cells. This is consistent with the presence of a VINE binding site in the C-terminal tail of Mrl1 but the uncertain nature of what this pattern represents makes the interpretation of the experiment unclear.

4. Whyte and Munro (PMID 11470415) presented evidence that Mrl1 helps sort proteinase B in yeast redundantly with Vps10 in SEY6210. Does SEY6210 also carry the vrl1 frameshift? If so, does this result suggest Mrl1 is capable of sorting proteinase B in the absence of Vrl1/Vin1? Could this assay be used to assess the role of VINE in the Mrl1-dependent sorting of proteinase B?

*Reviewer #2 (Recommendations for the authors):*

1. In the abstract, "has been overlooked due an inactivating mutation" should be "has been overlooked due to an inactivating mutation".

2. Pg. 3, line 90. I would disagree slightly that VARP is "the" mammalian homologue of Vrl1 given their significant structural and functional differences. The phrasing could be read to imply these two proteins are essentially orthologues. Perhaps it could be rephrased to simply say that the two proteins share homology in their RabGEF and AnkRD domains and have been found to share overlapping functions in endosomal Rab activation.

3. Pg. 5, lines 146-149. It is stated that Vrl1 lacks sidechains in its PX domain for lipid interaction. But it is then implied that the PX domain of Vin1 can bind to PI3P. After looking at its sequence and AlphaFold predicted structure it would appear that Vin1 also lacks residues necessary for phosphoinositide-binding. This should be clarified and discussed.

4. This is a slightly unusual suggestion and comment, but the emergence of AlphaFold2 lends itself to some novel ways of addressing interesting structural questions. Have the authors attempted to model the complex between Vrl1's AnkRD and the peptide region from the Vin1 N-terminus required for binding? I took the opportunity to attempt this myself using ColabFold and found that if I modelled the AnkRD domain with residues 70-100 of Vin1, that a consistent structure was predicted where residues 76-94 of Vin1 form a well-ordered α-helical structure bound to the N-terminus of the AnkRD structure (correlating with the experimental data in this manuscript). From experience modelling other peptide complexes I think this is a reasonably convincing model. The authors could easily perform this experiment and include the model themselves in their paper, and it may then be worth exploring with one or two single point mutations in the predicted interface using the established cellular membrane recruitment assays.

5. On the use of AlphaFold2, because the field is still in the early stages of establishing the best ways to report the data and estimating the accuracy of derived models we should take these early opportunities to report methods and results in as much detail as possible. The use of AlphaFold2 to test for potential BAR-BAR interactions is a really great use of the method, and Table S3 and Figure 3B provide an excellent summary of the systematic analysis. I don't think it's necessary to do this for all predicted pairs, but the predicted alignment error (PAE) plots could also be shown for some of the key examples. Although relatively qualitative, in our experience the off-diagonal regions of the PAE plot gives a very simple an accurate visual representation of when two domains or two proteins interact with each other. Similarly, the PAE plots for the predicted structures of Vrl1 and Vin1 could be provided as supplemental figures, as these will show clearly for example that the N-terminal region of Vin1 is disordered and not self-interacting, while for both proteins the PX and BAR domains are tightly coupled to each other structurally. Lastly, given there are now several Colab websites that can run AlphaFold2 with different variations the precise site used should be provided in the methods.

# Reply: Author response

**DOI:** 10.7554/eLife.77035.sa2

Essential revisions:

1) Resolve questions related to Mrl1 localization with experimental data and text clarification (see below)

We have carried out several new experiments to show that Mrl1 localizes to endosomes and not to lateral clusters in the vacuole membrane (see below). This supports our model that Vrl1 redistributes Mrl1 from endosomes, and – importantly – shows that restoring Vrl1 function selectively alters the endosomal localization of a recycling receptor. We recognize that proving VINE acts as a sorting complex by binding directly to a signal in the Mrl1 cytoplasmic domain would require additional experiments that are beyond the scope of this manuscript. As a result, we have now revised the text, title and abstract to ensure that our conclusions do not overstep our results. We have also further clarified our model and included alternative models in our discussion.

2) Model the complex between Vrl1's AnkRD and the peptide region from the Vin1 N-terminus, and test the model with a few mutants (see below).

We thank the reviewer for this excellent idea. We have now carried out these experiments (see below), and have added a full new figure (Figure 5) and two new supplementary figures (Figure 5 S1 and 5 S2) to our manuscript describing our results. We now present a model showing the binding of the Vin1^Nt^ critical region to a pocket in Vrl1^AnkRD^. We mutated several key residues in this predicted Vin1^Nt^-binding pocket and found that this either blocked or reduced recruitment of the Vin1^Nt^ using our quantitative chimeric recruitment assay. We feel that the addition of these new experiments has greatly strengthened the manuscript.

In addition, we have carried out other experiments, added a number of new supplemental figures (Figure 1 S1B and S1C; Figure 3 S1A, S1B and S2; Figure 4 S1; Figure 7 S1A and S1B) and made additional changes to the text in response to the reviewers’ comments, as detailed below.

Reviewer #1 (Recommendations for the authors):

My primary concern is with the Mrl1 localization data shown in Figure 6 and so most of my comments will focus on this issue.

1. The images presented for the vrl1 and VRL1 cells are very similar although the "GFP Puncta Per Cell" quantitation shows a strong reduction of puncta in cells with wild-type VRL1. A reduction in "bright" puncta is a subjective measure and could also be caused by lower expression levels of the MRL1-GFP or lateral changes in distribution around the vacuole membrane – not necessarily mislocalization from one organelle to another. The model in 6L argues for a role of VINE in sorting Mrl1 from endosomes to the Golgi, consistent with the function of other sorting nexins. If this is correct, I would expect cells with WT VRL1 would display a greater number of Mrl1-GFP puncta and the vrl1 mutant would display most of Mrl1 in the vacuole. The data shown in Figure 6C and D appears to indicate the opposite result. If the puncta are endosomes, the data would argue a role of VINE in the delivery of Mrl1 from endosomes to the vacuole. This is the conclusion stated in the abstract of the manuscript but the conclusion is reversed in the Figure 6L model. The authors should try to better define these Mrl1 puncta using less biased, more quantitative approaches to address potential changes in organelle localization. For example, do the Mrl1-GFP puncta co-localize with Did2-mRuby? Can the authors report a Manders coefficient between Did2-Ruby/Mrl1-GFP and Mrl1-GFP/Vph1-mCherry (vacuolar marker) in cells +/- Vrl1 that would support a VINE-dependent change in Mrl1 localization?

The idea that Vrl1 alters the lateral clustering of Mrl1 at the vacuole membrane, rather than redistributing it from endosomes, is interesting and one that we had not considered. To define the Mrl1 puncta, we used automated analysis to evaluate the colocalization of Mrl1-mScI with the endosomal markers GFP-Vps21 and Did2-Envy and the vacuolar membrane marker GFP-Ypt7*.* We observed strong overlap between the punctate Mrl1 signal and the endosomal markers, but not with the punctate Ypt7 signal at vacuoles, suggesting that the bright Mrl1 puncta represent endosomes and not clusters of Mrl1 on the vacuolar membrane. These data are presented in a new Figure 7 S1A and S1B and reported in the text (lines 280-283).

As suggested, we attempted a quantitative analysis to detect enhanced localization of Mrl1 to the vacuole in cells expressing Vrl1. However, we were not able to effectively measure Mrl1 signals around the vacuolar rim, which are weak relative to background. While it is straightforward to detect the loss of signal from endosomes (where it is concentrated in puncta), it is much harder to detect an increase in signal when it is redistributed around the much larger vacuolar surface (and thus diluted). For this reason, we have now softened our conclusion that Vrl1 causes more Mrl1 to appear on the vacuole membrane.

The presence of Mrl1 on the limiting vacuole membrane means that its signal is brighter at vacuole-vacuole contacts where two membranes are opposed. Our previous images inadvertently showed many such contacts, which obscured the differential localization at endosomes. We now provide representative micrographs for *MRL1-GFP vrl1* and *MRL1-GFP* p*VRL1* in Figure 7C that more clearly illustrate the reduction in puncta intensity at endosomes. As a further test, we evaluated the presence of Mrl1 at endosomes, vacuole-vacuole contacts or vacuole vertices in the presence or absence of *VRL1*. Author response image 1 shows that Vrl1 does not alter the localization of Mrl1 at vacuole-vacuole contacts or vacuole vertices, but clearly reduces the number of perivacuolar puncta that are typical of endosomes. Taken together, these data suggest that Vrl1 specifically prevents the accumulation of Mrl1 at endosomes.

**Author response image 1.** Vrl1 specifically redistributes Mrl1 from endosomes. Quantification of Mrl1-GFP localization in *vrl1* and *VRL1*-expressing strains. Blind scoring of GFP signal was conducted manually. Unpaired equal variance *t* tests; n = 3, cells/strain/replicate ≥ 465; not significant, n.s. = p > 0.05, * = p < 0.05. Scale bar, 2 µm. Error bars report standard error of the mean (SEM).

We present our favored model in Figure 7L (formerly Figure 6L) but agree that other interpretations are possible. Because the VINE complex forms a SNX-BAR coat which decorates budding endosomal structures and redistributes Mrl1 from endosomes, we feel it is reasonable to propose that it acts as a cargo sorting complex at the endosome. While it is logical to assume that VINE delivers cargo to the vacuole, there is currently no precedent for a SNX-BAR coat that mediates a direct endosome-to-vacuole transport pathway. Instead, our data suggests Mrl1 is primarily a vacuole membrane protein that may follow a recycling itinerary similar to that of Atg27 (Suzuki and Emr, 2018; PMID: 29511122). Atg27 is located at the vacuole membrane at steady-state, and maintains this distribution through the sequential action of Snx4 (which transports it from vacuole to endosome) and retromer (which recycles it from endosomes to the Golgi). Loss of retromer causes Atg27 to accumulate at endosomes, which mirrors the accumulation of Mrl1 at endosomes in vrl1 mutants.

We have made changes to the text to explain this model more clearly (lines 302-309). We realize that many aspects of this cargo sorting model are speculative, and other interpretations are possible. Therefore, we have made additional changes to the text, title and abstract to soften our conclusions that Mrl1 is a direct cargo of Vrl1 and propose alternative hypotheses.

2. Mrl1-GFP appears to be concentrated at sites of membrane contact between vacuoles (vertices) and possibly between vacuoles and the prevacuolar endosome compartment. Could the vacuole/prevacuolar contact site be the observed Mrl1 puncta? Co-localization with Ypt7 could test this possibility. Figure 1 reports an interaction between Vrl1 and Vps11/Vps18, part of the core HOPS complex which is found at these pre-fusion contact sites. It seems possible that VINE binding to HOPS and/or Mrl1 somehow prevents the concentration of Mrl1 at these contact sites and changes the lateral distribution of Mrl1 in the vacuolar membrane.

As described above in response to point 1, while some Mrl1 appears to be enriched at vacuole-vacuole interfaces (which could result from an increase in signal due to the two adjacent membranes), many Mrl1 puncta are at sites where no adjacent vacuole is present (see Figure 7E for example), and it is this population that appears to be preferentially affected by Vrl1. We also found that Mrl1 puncta showed little overlap with concentrated sites of Ypt7 (Figure 7 S1A and S1B), which suggests it is not enriched in prefusion sites. These results, together with those described above, have convinced us that Mrl1’s presence at endosomes (rather than lateral vacuole clusters vertices, or contact sites) is altered by Vrl1.

We agree it is interesting that our DHFR assay identified many endosomal proteins as proximal to Vrl1, including HOPS subunits. We do not yet know if this is meaningful, but we plan to investigate these interactions in future experiments.

3. Transplantation of the Mrl1 C-terminal tail onto Vps10∆CT caused a change in the localization pattern from uniform vacuole membrane with some concentration at vertices, to a localization pattern that looks more like Mrl1-GFP in the vrl1 cells. This is consistent with the presence of a VINE binding site in the C-terminal tail of Mrl1 but the uncertain nature of what this pattern represents makes the interpretation of the experiment unclear.

This experiment demonstrates that Vrl1’s effect on Mrl1 distribution can be mapped to its cytosolic domain. We agree that while this is consistent with the presence of a VINE binding site in the Mrl1 tail, other explanations are possible and Vrl1 could be indirectly affecting the sorting of Mrl1. Thus, we have been careful to add these caveats to our conclusions in the revised manuscript.

4. Whyte and Munro (PMID 11470415) presented evidence that Mrl1 helps sort proteinase B in yeast redundantly with Vps10 in SEY6210. Does SEY6210 also carry the vrl1 frameshift? If so, does this result suggest Mrl1 is capable of sorting proteinase B in the absence of Vrl1/Vin1? Could this assay be used to assess the role of VINE in the Mrl1-dependent sorting of proteinase B?

SEY6210 does carry the *vrl1* mutation. The work of Whyte and Munro suggests that Mrl1 is capable of sorting proteinase B in a VINE-independent manner, though the recycling pathways followed by Mrl1 were not clearly defined in that paper. Our tail transplantation experiment clearly showed that the Mrl1 tail has other sorting signals (Figure 7H and 7I), and papers from our lab (Bean et al., 2017; PMID: 27883263) and others (Best et al., 2020; PMID: 32074001; Suzuki et al., 2021; PMID: 34524084) have shown that different sorting nexins can act redundantly at endosomes. We have added new text to our Discussion section to account for the possibility that other sorting nexins may redundantly sort Mrl1, and have added a citation for Best et al., 2020.

Reviewer #2 (Recommendations for the authors):

1. In the abstract, "has been overlooked due an inactivating mutation" should be "has been overlooked due to an inactivating mutation".

This has been corrected.

2. Pg. 3, line 90. I would disagree slightly that VARP is "the" mammalian homologue of Vrl1 given their significant structural and functional differences. The phrasing could be read to imply these two proteins are essentially orthologues. Perhaps it could be rephrased to simply say that the two proteins share homology in their RabGEF and AnkRD domains and have been found to share overlapping functions in endosomal Rab activation.

The Dacks and Field labs have carried out a detailed evolutionary analysis of this gene family that shows the mammalian VARP and yeast Vrl1 are orthologs that trace their origins to the last eukaryotic common ancestor (Herman et al., 2018; PMID: 29603841). We have now included a reference to this paper to support our statement that these genes are orthologous (lines 100-102 in the revised manuscript).

3. Pg. 5, lines 146-149. It is stated that Vrl1 lacks sidechains in its PX domain for lipid interaction. But it is then implied that the PX domain of Vin1 can bind to PI3P. After looking at its sequence and AlphaFold predicted structure it would appear that Vin1 also lacks residues necessary for phosphoinositide-binding. This should be clarified and discussed.

We have added a short discussion of this on line 154-156 including reference to a recent study that finds that the Vin1 (therein named Vps501) PX domain recognizes PI3P through an atypical motif (Goyal et al., 2022; PMID: 29603841). Yu and Lemmon (2001; PMID: 11557775) also report that purified Vin1 binds to PI3P specifically in vitro, as mentioned on lines 134-135.

4. This is a slightly unusual suggestion and comment, but the emergence of AlphaFold2 lends itself to some novel ways of addressing interesting structural questions. Have the authors attempted to model the complex between Vrl1's AnkRD and the peptide region from the Vin1 N-terminus required for binding? I took the opportunity to attempt this myself using ColabFold and found that if I modelled the AnkRD domain with residues 70-100 of Vin1, that a consistent structure was predicted where residues 76-94 of Vin1 form a well-ordered α-helical structure bound to the N-terminus of the AnkRD structure (correlating with the experimental data in this manuscript). From experience modelling other peptide complexes I think this is a reasonably convincing model. The authors could easily perform this experiment and include the model themselves in their paper, and it may then be worth exploring with one or two single point mutations in the predicted interface using the established cellular membrane recruitment assays.

We thank the reviewer for this very thoughtful suggestion. We have now added a full figure (Figure 5, 5 S1 and 5 S2) to our manuscript with results from modelling the interaction between Vrl1^AnkRD^ and Vin1^Nt^. We also mutated the predicted Vrl1 Vin1^Nt^-binding pocket in the context of our Vrl1(1-703)^YPE^ chimera and found that this either blocked or reduced recruitment of the Vin1^Nt^ depending on which mutations we made. We believe that these suggests allowed us to greatly improve our manuscript, and we are grateful for them.

5. On the use of AlphaFold2, because the field is still in the early stages of establishing the best ways to report the data and estimating the accuracy of derived models we should take these early opportunities to report methods and results in as much detail as possible. The use of AlphaFold2 to test for potential BAR-BAR interactions is a really great use of the method, and Table S3 and Figure 3B provide an excellent summary of the systematic analysis. I don't think it's necessary to do this for all predicted pairs, but the predicted alignment error (PAE) plots could also be shown for some of the key examples. Although relatively qualitative, in our experience the off-diagonal regions of the PAE plot gives a very simple an accurate visual representation of when two domains or two proteins interact with each other. Similarly, the PAE plots for the predicted structures of Vrl1 and Vin1 could be provided as supplemental figures, as these will show clearly for example that the N-terminal region of Vin1 is disordered and not self-interacting, while for both proteins the PX and BAR domains are tightly coupled to each other structurally. Lastly, given there are now several Colab websites that can run AlphaFold2 with different variations the precise site used should be provided in the methods.

We have now included ColabFold-generated PAE plots for each individual sequence query (Figure 1 S1C and Figure 4 S1) and for each binding prediction (Figure 3 S1B and Figure 5 S1B), including boxes denoting various predicted domains or regions of disorder. We have also included pLDDT-colored models of our predictions (Figure 1 S1B, Figure 3 S1A, Figure 5 S1A and Figure 5 S1C).

We also used the PAE plots in Figure 3 S1B to make comparisons between pairs of SNX-BARs that were predicted to interact by our parameters (ie Vrl1 and Vin1) and pairs of SNX-BARs that were not predicted to interact (ie Vrl1 and Vps17).

We have included more detailed information about which version of the ColabFold program was used, including a link to the notebook, as well as the updated citation for ColabFold (Mirdita et al., 2022; PMID: 35637307).
